# Retrospective Evaluation of Sequential Events and the Influence of Preference-Dependent Working Memory: A Computational Examination

**DOI:** 10.3389/fncom.2020.00065

**Published:** 2020-09-11

**Authors:** Sewoong Lim, Sangsup Yoon, Jaehyung Kwon, Jerald D. Kralik, Jaeseung Jeong

**Affiliations:** Department of Bio and Brain Engineering, Korea Advanced Institute of Science and Technology (KAIST), Daejeon, South Korea

**Keywords:** decision making, working memory, retrospective evaluation, cognitive heuristics, peak-end effect

## Abstract

Humans organize sequences of events into a single overall experience, and evaluate the aggregated experience as a whole, such as a generally pleasant dinner, movie, or trip. However, such evaluations are potentially computationally taxing, and so our brains must employ heuristics (i.e., approximations). For example, the peak-end rule hypothesis suggests that we average the peaks and end of a sequential event vs. integrating every moment. However, there is no general model to test viable hypotheses quantitatively. Here, we propose a general model and test among multiple specific ones, while also examining the role of working memory. The models were tested with a novel picture-rating task. We first compared averaging across entire sequences vs. the peak-end heuristic. Correlation tests indicated that averaging prevailed, with peak and end both still having significant prediction power. Given this, we developed generalized order-dependent and relative-preference-dependent models to subsume averaging, peak and end. The combined model improved the prediction power. However, based on limitations of relative-preference—including imposing a potentially arbitrary ranking among preferences—we introduced an absolute-preference-dependent model, which successfully explained the remembered utilities. Yet, because using all experiences in a sequence requires too much memory as real-world settings scale, we then tested “windowed” models, i.e., evaluation within a specified window. The windowed (absolute) preference-dependent (WP) model explained the empirical data with long sequences better than without windowing. However, because fixed-windowed models harbor their own limitations—including an inability to capture peak-event influences beyond a fixed window—we then developed discounting models. With (absolute) preference-dependence added to the discounting rate, the results showed that the discounting model reflected the actual working memory of the participants, and that the preference-dependent discounting (PD) model described different features from the WP model. Taken together, we propose a combined WP-PD model as a means by which people evaluate experiences, suggesting preference-dependent working-memory as a significant factor underlying our evaluations.

## Introduction

Cognitive psychologists and behavioral economists have uncovered various heuristics (i.e., approximations) that the human brain uses to resolve the curse of dimensionality (i.e., too much information in the natural world to process fully). Heuristics are akin to visual illusions that provide a window into how the brain actually processes information. One of the noted problems studied is how people evaluate sequences of events, such as the overall pleasantness of a dinner, movie, or trip. This organization of singular moments into larger events is perhaps deceptively simple, yet is one of the fundamental capabilities underpinning human higher cognition (e.g., learning, memory and decision-making). For example, in decision-making, what we experience influences future choices, as we attempt to pursue good experiences (based on expected value learned and remembered from previous experiences) and avoid bad ones. How we evaluate these experiences thus critically influences future decision-making. And yet the evaluation of aggregated experiences is not simple, for it could entail integrating the pleasure across every moment of the experience, which is not normally feasible.

How then does the human brain evaluate past experiences? The overall assessment would be expected to lie somewhere between the highest and lowest values of the individual moments of the experience, but there are many possibilities, and so we may ask, which of them does the brain actually use? That is, does it have a preferred approach? Indeed, it has been shown that people appear to focus more on the peaks of a sequential event, such as the pain of an aversive experience, as opposed to its duration, leading to a bias in which reducing the *peak* of the pain is preferred to lessening the *duration* of pain, resulting in duration neglect (Varey and Kahneman, 1992; Fredrickson and Kahneman, 1993). For example, when choosing between one option of holding one's hand in a bucket of freezing cold water for a particular duration, e.g., 14°C for 60 s, and another option of 14°C for 60 s plus an additional 30 s at 15°C, i.e., slightly less cold, people prefer the latter option, even though it results in 50% more pain (90 vs. 60 s). The experience-evaluation model proposed for such phenomena has been presented as a *peak-end rule*, which suggests that the peak of an experience and the most recent experience make the greatest contribution to the experience assessment (Kahneman et al., 1993).

Since discovering this phenomenon, there has been active interest in examining sequence evaluation and determining exactly when and if the peak-end rule is in fact utilized, with many studies testing the evaluation model under various conditions (Langer et al., 2005; Rode et al., 2007; Do et al., 2008; Kemp et al., 2008; Liersch and McKenzie, 2009; Legg and Sweeny, 2013; Xiaowei et al., 2013). The peak-end rule has been found to hold for some cases, but not for others. For example, it appears to hold for studies with material goods (Do et al., 2008) and a news-giving situation (Legg and Sweeny, 2013). In a meal study, however, duration neglect and a preference for patterns rising in likeability were found even if the peak-end bias was not (Rode et al., 2007). A survey study of vacationing as a several-day-scale experience showed that the peak-end rule was not an outstandingly good estimator (Kemp et al., 2008). Other studies have tried to determine the conditions under which the peak-end rule holds (Langer et al., 2005; Liersch and McKenzie, 2009; Xiaowei et al., 2013). In one study, the peak-end bias disappeared when the stimulus was simply presented without any distraction (Langer et al., 2005). In another, a numerical stimulus showed peak-end bias but a graphical stimulus did not (Liersch and McKenzie, 2009). Moreover, both Langer et al. (2005) and Liersch and McKenzie (2009) showed that in easy tasks, without distractions or need for high memory capacity, performance did not follow the peak-end rule. The evaluation rule also changed based on the timescale of the experience, only seeming to hold for short retention intervals (Xiaowei et al., 2013). These results suggest a potentially significant role played by working memory in the sequence-evaluation process (Miller, 1956; Cowan, 2001). There were also studies of the peak-end rule with non-human primates, but the results were mixed (Xu et al., 2011; Jung and Kralik, 2013; Blanchard et al., 2014).

Experience evaluation patterns have been examined in other fields that require decision making based on evaluation of a series. In a study on dynamic pricing problems, the peak-end rule was assumed to estimate the reference price for a pricing model (Nasiry and Popescu, 2011). In a study on learning experiences, the researchers tried to determine the type of study experience that makes it easier to learn and found that the peak-end rule held in the experiment (Hoogerheide and Paas, 2012). Such diverse possible applications of the rule have spawned many reviews that attempt to define the problem and suggest evaluation models and experimental designs (Kahneman et al., 1997; Ariely and Carmon, 2000; Fredrickson, 2000; Kahneman, 2000).

Defining *utilities* with respect to experiences, peak-end evaluation is understood as a rule to predict *remembered* utility, as distinguished from *decision* utility or *total* utility (Kahneman et al., 1997). Remembered utility is obtained from subjective survey after the event of interest (e.g., after the entire series composing the event is experienced), and total utility is a theoretical value computed from a series of experiences, assuming a model to compute the representative of the series. The peak-end rule is a kind of evaluation by moments, with the idea of “judgment by prototype” moment(s) (Kahneman, 2000). It says that an evaluation is determined primarily by some representative samples. *Peak* and *end* are interpreted as carriers of meaning, and *experience evaluation* is extraction of meaning (Fredrickson, 2000). Nonetheless, peak and end may not be the only characteristics in experiences that could be representative features (Ariely and Carmon, 2000). And even then it can be difficult to differentiate among possible representative characteristics in a given setting. For example, a study showed that the peak-end rule and simple averaging of experiences coincide if the moment utility model has individual heterogeneity and perceptual persistence (Cojuharenco and Ryvkin, 2008). Moreover, there are methodological considerations yet to be fully addressed, such as types of experiences, positivity/negativity of experiences, timescale of experiences, quantitative/qualitative experiences, and real/virtual experiences. Selective attention and memory mechanisms are also proposed as possible encoding-based explanations (Ariely and Carmon, 2000), although the correlation between the peak-end rule and working memory remains unclear.

To address these myriad outstanding issues, it will require a comprehensive quantitative assessment, and yet to our knowledge, to date there is no precise experience evaluation model to do so. For example, how much do peak and end truly contribute to the evaluation? What about the second peak or just before “end”? The current study was designed to address this general limitation.

In particular, there were two main goals of the current study. The first was to develop an evaluation model that best represents the specific mechanisms underlying sequence evaluations; and the second was to determine how working memory is involved in the evaluations. For these purposes, we also designed experiments that measure the pattern of experience evaluation and the working memory ability involved in experience evaluation.

To determine how sequences are evaluated, and more specifically, how the temporal length of experience affects evaluation, certain types of experimental data are needed. We therefore conducted several behavioral experiments: a *picture-rating task*, a *sequence-rating task* of four and seven pictures with a simultaneous *working memory task*, and a *sequence-rating-continued–version task* for 100 pictures. The first three tasks (and data collected) were also utilized in a related study in our laboratory that focused on a different phenomenon in decision dynamics known as serial choice: how people decide which sequence of items they will choose in the future, e.g., whether we prefer selecting the best items first or save them for last (Yoon et al., 2019). In that study, the experimental question was focusing on serial choice and comparing it to several other related phenomena like delay discounting, sequence rating and working memory to determine the extent to which serial choice was a separable phenomenon in its own right. The sequence rating and working memory task data were only examined in that study to test whether they correlated with serial choice, which they did not. The current study on experience evaluation addressed an entirely different set of questions, focusing on computational modeling to uncover the specific factors underlying sequence rating, as well as its potential relation to working memory. Again, the current study also used a novel sequence-rating-continued-version task to measure longer sequences of unspecified lengths.

After completion of the empirical experiments (i.e., the set of tasks), we then proposed four basic models including a peak-end model and averaging model (averaging all experiences for total evaluation), as well as *peak* alone and *end* alone, to test whether the peak-end rule hypothesis held or not, and the extent to which each element may underlie the sequence evaluations. The averaging model actually proved most effective, though the results also provided evidence for multiple influences, leading us to develop a more general model that examined averaging in combination with order and preference dependence. The subsequent model successfully correlated with the experimental results, but ultimately proves infeasible in that it requires all experiences to be remembered regardless of sequence length. Inspired by these initial results, yet in pursuit of a more plausible account with greater generalizability, we then introduced windowed-evaluation models (i.e., evaluation of events within a specified time window from the last experienced moment into the past) that highly correlated with the empirical results. These models, however, still left significant variance unaccounted for, likely stemming from inherent weaknesses of window-based models, such as ignoring potential lasting effects of particularly salient individual events outside of the evaluation window. We then introduced discounting models to account for these longer-lasting effects, which indeed appeared to capture these effects. In addition, a comparison of the temporal-discounting rates with the working-memory data showed that the working-memory capacity of individual participants correlated with temporal discounting, providing validation for temporal discounting underlying the sequence ratings, and evidence for preference-dependent working-memory as a significant factor underlying our evaluations. Given that a windowed preference-dependent model as well as a discounting preference-dependent model each appeared to account for different characteristics of the data, we ultimately found that a combination of the two models together provided both the highest explanatory power as well as greatest flexibility to capture individual differences in retrospective evaluation. Finally, we emphasize that it is important to report this systematic progression from the initial hypotheses (e.g., testing the peak-end rule) to the best-fitting model, to clarify exactly what factors and to what degree they successfully explain the sequence evaluations, as well as the extent to which other components fall short.

## Experiments

As an experience to be properly evaluated, it was critical to use a real and immediately consumable reward, that maintained a reliable, sustained effect across the entire study. Faces and images of people are easily recognized and remembered, particularly for those of the opposite gender. In addition, there is neuroimaging evidence that facial expressions affect the reward system of the brain (O'Doherty et al., 2003; Mühlberger et al., 2010). Moreover, evolutionary considerations validated by multiple studies based on both behavioral and neurobiological assays have shown that visual images of the opposite gender are rewarding; and this is especially so for heterosexual men viewing women (Aharon et al., 2001; Hamann et al., 2004; Hayden et al., 2007; Rupp and Wallen, 2007; Cloutier et al., 2008; Stevens and Hamann, 2012). And as such, we would expect that findings based on attractiveness would generalize to other types of reward. Also, as image data, pictures are more easily classified and standardized by size, color, brightness, and so on, as well as accessed and searched for as publicly available data. For all these reasons, we tested male participants with pictures of the opposite gender (female) as the experimental stimuli to achieve our research aims. Once the paradigm were established, a comparable study with female participants could then be conducted.

The Institutional Review Board (IRB) of KAIST approved all experimental procedures for this study. We tested 66 male participants (self-reported heterosexual, age 23.42 ± 3.06) in a 3 day experiment. Informed written consent was obtained from all participants. On the first day, the picture-rating task was performed to measure preferences for a set of pictures to be used in later tasks. In addition, participants also performed the “sequence-rating-continued-version task.” On the second day, the picture-rating task was performed again to produce robust and reliable picture-rating data for the subsequent experiments. On the third day, the sequence-rating and working-memory combined task with four pictures was conducted. After that, the same sequence-rating and working-memory task was conducted with seven pictures in a sequence.

As noted above, the data from the picture-rating, sequence-rating, and working-memory tasks were also used in a different study by our laboratory that focused on serial choice, i.e., the preferred order people have when choosing a sequence of items, whether, for example, preferring to experience their favorite first or last (Yoon et al., 2019). That study found no correlation between serial choice and the retrospective evaluations, suggesting that both were separate phenomena. The current study focuses on the specific mechanisms underlying retrospective evaluation. In the descriptions below, all text displayed to the participants was in Korean (translated here in English).

### Preparing Picture Set

To isolate attractiveness as best as possible, we collected images from Google image search (https://images.google.com/) with keywords “Asian,” “woman,” “girl,” “bikini,” though many had various types of clothing. We selected pictures containing only a single subject with a clearly visible face and eye gaze. We excluded pictures with texts, animals or any emotionally salient objects like food, weapons, or luxury items. Pictures which were small or blurry or had a clear expression of negative emotion or appeared to be younger than 19 years old were also excluded. By this process, we collected a robust set of pictures (shared with Yoon et al., 2019) that should have provided a range of attractiveness levels of the women (from this evolutionary mating systems perspective).

Even though the attractiveness ratings might be expected to be similar across participants (agreeing on which images were more and less attractive), we wish to emphasize that any similarity across participants was not necessary for our study. That is, our study focused on how one's individual preferences for experiences—the specific pictures—are related to the preferences for a *sequence* of pictures. Thus, it in fact did not matter whether preferences were shared among the participants, only whether we could obtain reliable preferences within participants, which we did, as described next.

### Picture Rating

It was first critical to validate our novel experimental paradigm by obtaining reliable ratings of pictures that reflected actual rewarding experiences. We used the picture-rating task to do so (in both the current study and in Yoon et al., 2019). A prepared set of 500 pictures was divided into 10 subsets, 50 pictures each, where each subset corresponded to one session of the picture-rating task. One session consisted of 50 trials that first present a picture for 1.3 s then asks the preference of that picture. The query to “Evaluate the attractiveness of the picture” was displayed on the screen (in Korean) along with a horizontal 1–9 range scale bar below the text. If the participant pressed a number key on the keyboard, that number on the scale bar was changed to blue. If he pressed the enter key, the evaluation was confirmed. Before pressing the enter key, he could freely change the active (blue) number on the scale bar without a time limit. Response time for answers was 2.5395 s on average, with standard deviation 0.9513 s. After the attractiveness evaluation, the participant was asked the familiarity of the picture. Prior to the start of the task, the initial instruction “If you know the person in the picture or you have seen the picture, check yes. Otherwise check no.” was given. During the task, the question “Did you know the person in the picture?” was displayed on the screen with yes/no choices for each picture. The participant could check yes/no by using the left/right arrow keys. After each trial finished, there was a 1–3 s inter-trial interval.

The task was performed twice with a 1 week interval. On the second day, the picture-rating task only queried attractiveness since familiarity was determined on the first day. Upon completion of the second day session, each picture had two evaluations (attractiveness in 1–9 range) and familiarity (whether he knows the person or has seen the picture). To avoid using familiar pictures, only those with familiarity “no” were subsequently used for the remainder of the study. The two evaluations were then averaged for representative attractiveness, and thus the “picture rating.” Averaging two integers (1–9) produced picture ratings in the 1–9 range with 0.5 interval: 1, 1.5, 2, 2.5, …, 8.5, 9. The first and second day ratings were correlated with Pearson correlation 0.7458 on average with standard deviation 0.0782.

The picture-rating task generated a set of pictures for each participant, smaller than or equal to 500, with corresponding ratings. The pictures were sorted by their ratings and split into seven partitions for each participant, with boundaries determined by counting pictures for each rating value to make each partition evenly allocated. Partitions were labeled “1,” “1.5 star,” …, “3.5,” “4 star.” Furthermore, when generating partitions, the pictures for the half intervals (“1.5,” “2.5,” “3.5 star”) were assigned as small as possible, and the sizes of the full intervals (“1” – “4 star”) were made similar. The four integer groups “1” – “4 star” were used in all tasks, whereas the additional three groups “1.5 star” – “3.5 star” were used in later tasks with sequence length seven only. We minimized the use of these intermediate groups, to promote larger differences across the groups. The sizes of the seven partitions were 84.3182 ± 14.5223, 39.9394 ± 15.1352, 91.6061 ± 17.6686, 44.1212 ± 14.6639, 91.3788 ± 17.2785, 42.0758 ± 13.7060, 86.1970 ± 16.2426, respectively, averaging for participants ± standard deviations.

From the picture-rating task, the rating for each picture was defined as the *subjective rating* of the participants. Pictures with similar preferences were grouped together as described above. The individual picture ratings themselves were used by the models as independent variables, while the grouping was used only in measuring the preference-dependent working memory ability in section Discounting Models (Discounting rates and working memory).

### Sequence Rating and Working Memory

The experimental procedure (consisting of both the sequence-rating and working-memory tasks) consisted of 10 sessions (9 sessions of 15 trials and one session of 11 trials) to obtain 96 trials for sequence ratings and working-memory queries. The inter-trial interval was 2–5 s. In each trial, the instruction “Four pictures will appear soon. Look at the pictures carefully.” was displayed on the computer screen for 2 s and then four pictures were presented sequentially for 1.3 s each. The query “Evaluate your overall satisfaction of the four pictures.” was then displayed on the screen with the same scale bar as the picture-rating task. The procedure for entering numbers and using the enter key was also the same as the picture-rating task.

Then, for the working-memory task component, one of the previous four pictures appeared with the following question below it: “In which position of the sequence was this picture?,” along with a scale bar indicating from “1st” to “4th,” with the participants using the 1–4 number keys. This second query appeared at least 5 s after the previous four pictures were shown, to prevent the participant from not answering the first question carefully.

In addition, the same type of task with a sequence length of seven was conducted with 36 trials. For the sequence-rating tasks, the sequence rating for each sequence containing four or seven pictures was defined as the *subjective rating* of the sequence by the participants, with the sequence length defined as 4 or 7.

As described, the working-memory task was interleaved with the sequence-rating task with sequence of length 4, as participants were required to remember the position of each of the four pictures in the sequence. With only four images in the sequence, one might question whether the task sufficiently tested working memory, especially in light of well-established findings that suggest four and seven items should be comfortably within our short-term memory capacity (Miller, 1956; Cowan, 2001). Nonetheless, our results showed the participants' working memories were challenged by our task. As reported below (and in Yoon et al., 2019), their accuracy rate was between 0.55 and 0.95, meaning that for many participants it was not easy to maintain four pictures in working memory in the simultaneous sequence-rating setting. In fact, we originally piloted the working-memory task to verify that participants' working memory was sufficiently taxed.

### Sequence Rating Continued Version

For the sequence-rating and working-memory tasks, one could potentially memorize the entire sequence in a trial. This would often not be usual in real life and thus might unrealistically influence sequence ratings and diminish working-memory effects in the study. To avoid it, we also conducted a sequence-rating task with sequences of 100 pictures, which could not be memorized.

This task consisted of five sessions, 10 trials each. One hundred pictures were presented on the screen one by one in each session, and the participants performed a total satisfaction evaluation at random intervals of 8 to 13 pictures, making 10 pictures on average and a total of 100 pictures with 10 trials. For each trial, the participants entered the total satisfaction of all pictures the participant viewed from the beginning of the session to that time. For example, a participant might see 10 initial pictures, then be asked to evaluate the 10-picture sequence; then the participant observes eight more, then again is asked to rate the entire 18-picture sequence, and so on. It was made clear by instruction that the answer (i.e., satisfaction evaluation) on the last trial was an evaluation of all 100 pictures. Each picture was shown for 1.3 s, and participants entered a number ranging from 1 to 9.

From the sequence-rating-continued-version task, the sequence rating for each sequence of different lengths across the task was defined as the *subjective rating* of the participants. The sequence length was 8 at minimum (the earliest we could have queried them) and 100 at maximum (the final time we queried them), indicating the number of pictures participants had seen before rating.

In sum, we utilized two types of sequence-rating tasks: the sequence-rating task proper and the sequence-rating-continued-version task. The sequence-rating task, which was conducted simultaneously with the working-memory task, used two fixed lengths of sequences: lengths of 4 and 7. The sequence-rating-continued-version task used sequences without a fixed length. In this task, 100 pictures were sequentially presented, with the participants providing their current running evaluation of the entire sequence each time we queried them in 8–13 picture intervals. The final objective sequence length then was 100 pictures, whereas their subjective sequences (i.e., the actual number of pictures participants used to make their evaluation) could have been much shorter.

### Data Acquisition

In the picture-rating task, the average preference score of the two responses from each session was obtained for each picture. Only the pictures without familiarity were used in the subsequent tasks (i.e., assessed and removed for each participant individually), to exclude, for example, differences in memory due to familiarity in the working-memory task. The sequence-rating task asked two questions. The first measured the *satisfaction evaluation* corresponding to each sequence composed of four or seven pictures. The second question tested whether the participants could remember the correct order of a particular queried picture (i.e., position of the picture in the presented sequence), and we computed correct answer rates in three types: order-dependent, preference-dependent, and total correct rates. The sequence-rating-continued-version task measured the satisfaction evaluation corresponding to each sequence composed of 8 to 100 pictures. Note that the length of the sequence was not fixed in this case.

## Models

In this section, we explain the mathematical framework of our models and analyses. We attempted to use the simplest models possible to test the hypothesized factors underlying sequence rating, and we then progressively built additional models based on the results (i.e., the tests of the models against the empirical data). Here, we define the components of the evaluation models including stimulus, sequence and utilities, and the evaluation model itself. We also describe how the models were tested.

Stimulus *s*_*t*_ at time *t* represents the picture displayed at that moment. The participant then converts it into moment utility *x*_*t*_, which has a real number. When the participant is asked to evaluate whole experiences, the total utility is defined as *y*(*t*) = *U*(*x*_1_, *x*_2_, ⋯ , *x*_*t*_). No persistency effect (i.e., the effect of previous pictures on current picture ratings) is assumed in the picture-rating task and *x*_*t*_ is directly taken from the picture-rating task. To reduce any persistency effect, the picture-rating task was conducted twice for each picture, on separate days (roughly 7 days apart), in different order, and then averaged together. From this task, we obtained reliable picture ratings that we used as the picture rating values for *x*_*t*_, i.e., the *moment utilities*, of the pictures making up a sequence viewed in the sequence-rating tasks. Total utility *y*(*t*) is the output of the model to predict the rating of the sequence (remembered utility, explained in the Introduction), the value that the participant chose for the sequential experience in the corresponding sequence-rating task. The goal of the models in this study, then, is to generate total utility *y*(*t*) that matches the sequence ratings of the participants measured in the tasks.

### Total Utility Models

In the description above, the function from a sequence {*x*_*i*_} to the total utility *y*(*t*) is the *evaluation model* in this study:

(1) $$\begin{array}{l} y\left(t\right)=f\left(x_{1},x_{2},\cdots \;,x_{t}\right) \end{array}$$

Evaluation models are independent of the remembered (empirical) utilities and they can be any kind of functions generating their own total utilities. The main goal of our study was to find an evaluation model that generates total utilities similar to the remembered utilities of the participants, thereby using it to understand how people evaluate experiences.

All models in this study have the above form of Equation (1), including the peak-end, windowed-evaluation and discounting models. Some example cases are introduced here, but detailed analyses are in the later sections. The simplest version is taking the average of the entire sequence:

(2) $$\begin{array}{l} y_{\text{avg}}\left(t\right)=\frac{1}{t}\underset{1\leq i\leq t}{\sum }x_{i} \end{array}$$

We also tested the peak-end rule with the form:

(3) $$\begin{array}{l} y_{\text{PE}}\left(t\right)=\frac{1}{2}\left[\underset{1\leq i\leq t}{\max}\{x_{i}\right\}+x_{t}] \end{array}$$

## Test for Total Utility Models

To test how well each model accounts for the experimental data, we used Pearson's correlation analysis:

(4) $$\begin{array}{l} \rho_{\text{model}}=\text{corr}\left(y_{\text{model}},\;y\right) \end{array}$$

It has a value between −1 and 1 with a high correlation obviously meaning the model prediction is similar to the responses in the experiment. The variable *y* is the actual remembered utility of the participants: i.e., the rating of a sequence viewed in the two types of sequence-rating tasks (sequence-rating and sequence-rating-continued-version tasks). The variable *y*_*model*_ is the total utility of a sequence as computed by the sequence evaluation models: i.e., the prediction of the participants subjective ratings (actual remembered utility). The variable ρ_*model*_ measures how closely the model's prediction matches the actual participants' remembered utility.

For model parameter optimization, there are several measures to potentially use: a distance measure like mean squared errors (MSE), correlation measures like Pearson's correlation, Spearman correlation and Kendall correlation, and distributional divergence like Kullback-Leibler divergence (KL divergence). This study used Pearson's correlation as the criterion for optimization for several reasons.

First, we are solving the regression problem in a continuous target domain, and we are not assuming probabilistic distributions on evaluation or estimation. To use KL divergence in our problem, the target (remembered utility) must be assumed as a distribution (sharp Gaussian, for example) and we need to construct a robust histogram of all possible sequences. We would need additional hyper-parameters of the Gaussian variance of the target values and the number and size of bins to build the histograms. This is not feasible in our setting because the domain of the histogram has dimension of the number of independent variables, and it is the length of the sequence, 100, in the sequence-rating-continued-version task. Moreover, the distributional loss in the regression problem helps to reduce the steps for optimization rather than preventing overfitting or improving the representation (Imani and White, 2018). We did not have any convergence or learning speed problem in the study, so we did not use the KL-divergence as a criterion for optimization.

Second, correlation measures are scale-free measures of loss. In our experimental design, independent variables are picture ratings and the dependent variable is the sequence rating. In principle, the two types of ratings are in different units. Sequence ratings and picture ratings were measured in different experiments by subjective ratings. The two experiments are independent and each utility can be defined by other means (not ratings): For example, we can define the remembered utility by EEG signals rather than subjective ratings where y has a unit of Volt. The two variables are thus in different domains, and the total utility predicted by the evaluation model, which should be compared to the remembered utility, is computed on the domain of moment utilities. For example, if a model predicted sequence ratings “0.5, 1, 1.5, 2” for four different sequences, if the participant rated the sequences as “1, 2, 3, 4,” then the model provides a perfect prediction as an experience-evaluation model. Moreover, in the scale-free situation, minimizing MSE of the re-scaled prediction is equivalent to maximizing the absolute Pearson's correlation. That is, if the model can be written as *y* = *f*(*x*; *r*), with parameter set *r*, then minimizing MSE(*y*_*i*_, *a* • *f*(*x*_*i*_; *r*) + *b*) over *a, b*, and *r* is equivalent to maximizing |corr(*y*_*i*_, *f*(*x*_*i*_; *r*))| over *r*. This is because when we minimize the MSE, we can first determine a and b as functions of *x*_*i*_, *y*_*i*_ and *r* by simple linear regression since there are no interaction terms between *a, b*, and *r*. Then MSE can be expressed only by *x*_*i*_, *y*_*i*_,, and *r*, and it gives the relation $\frac{\text{MSE}}{s_{y}^{2}}=1-{\text{corr}}^{2},$ where $s_{y}^{2}$ is the variance of *y*_*i*_. If we minimize MSE of a re-scaled prediction, then the absolute correlation is also maximized. Because of these reasons, we used Pearson's correlation as the criteria for the optimization. Linear models, the preference-dependent model and the windowed models satisfied the condition so their re-scaled MSEs are also minimized. Discounting models, which should be understood as *f(x;r)* in the above notation, actually optimized MSE between the target and re-scaled model prediction. Therefore, we are consistently minimizing MSE of the re-scalable models. Thus, we used Pearson's correlation as the representative measure for optimization.

Third and finally, correlation measures like Spearman and Kendall correlations test only for the monotonicity of the relation. For this study, we were interested in a more rigorous test of the models against the actual sequence ratings of the participants by providing the best fits that actually minimized MSE of the re-scaled predictions. Nonetheless, additional measures help validate the robustness of the results, and thus we also computed Spearman and Kendall correlations together with Pearson's correlation to examine the robustness of our findings. A comparison of the correlations showed that the results did not change based on the correlation used. We thus only report the computed Spearman and Kendall correlations for representative models in the final results section rather than using them as additional criteria of optimization.

### The Specific Models in the Study

Since our study sought to determine exactly how the human brain evaluates sequences of events as one integrated unit, we considered several models based on the weighted sum formula, *y* = ∑ *w*_*i*_*x*_*i*_. We started from the four models—Peak, End, Peak-End, and Averaging—to test the hypothesis that the peak-end rule predicts better than averaging all events in the sequence. These simple models have no parameters, thus no need to be learned, and use different weightings *w*_*i*_ for each event in the sequence. As reported in the following section, the results on sequences with length four and seven show that not only does Averaging better explain the actual sequence ratings than does Peak-End, but they also show that the participants are doing more than just averaging all of the moment utilities. Based on this finding, in section Relative-Preference-Dependent and Order-Dependent Models we next attempted to generalize the four models to multivariate linear models, where Peak and End receive different indexing for *x*_*i*._ We compared two weights with the different indexing—relative-preference-dependent and order-dependent weights—which are generalizations of the Peak and End, respectively; and we combined them as a generalized Peak-End-Averaging model. As shown in section Relative-Preference-Dependent and Order-Dependent Models, the results show a greater degree of matching with the empirical results (i.e., higher correlation).

Although the model in section Relative-Preference-Dependent and Order-Dependent Models showed a significantly high correlation with the actual sequence ratings of the participants, it nonetheless has a fundamental limitation: it parametrizes all weights corresponding to the moment utilities, with the number of parameters the same as the length of the sequence. It can be applied to the sequences with length four or seven, but cannot be applied to sequences in the sequence-rating-continued-version task, whose lengths are not fixed and up to 100. A different form of parametrization was thus required to solve this problem. First, in section Absolute-Preference-Dependent Model we found that an absolute-preference-dependent model successfully replaced the relative-preference-dependent model. We note that this change made the model non-linear due to the dependence of weights *w*_*i*_ on the moment utilities *x*_*i*_; and thus, the models in sections “Absolute-Preference-Dependent Model,” “Windowed Evaluation,” and “Discounting Models” are non-linear. Second, to further address the scaling problem, in section Windowed Evaluation we tested windowed models (i.e., evaluation of events within a fixed time window) and found evidence for the use of such windows in the participant ratings. At the same time, a fixed window of sequence evaluation appeared to miss some aspects of the results: in particular, the influence of particularly salient events that fall outside of the window. In section Discounting Models, then, we tested Discounting models to potentially capture this effect, and found evidence that they in fact did so. Finally, also in section Discounting Models, after further examination of the results in light of the Windowed and Discounting model fits, it appeared that both models were indeed capturing different aspects of the data. This was then confirmed by the increased performance of our final model, a combination of both the absolute-preference-dependent windowed model and the absolute-preference-dependent discounting models to capture order dependence together.

To be clear, for parameter fitting of the models, we note that every model parameter (if the model had any parameter) was determined by an optimization process, normally via maximizing the Pearson's correlation. For example, the averaging and peak-end models have no parameter so they can be directly applied to predict sequence ratings. The order- and relative-preference-dependent models have 4 parameters with sequence length 4, so the four parameters are determined by maximizing the Pearson's correlation (technically minimizing MSE based on their linearity). Parameters for the other models were also determined in the same way, and this consistent criterion does not impair earlier model settings. Moreover, the optimization was not only performed for parameter assignments of the linear models. For optimization of the non-linear models, the windowed models used a parameter sweep when determining the window sizes, and the discounting models used gradient descent to determine the discounting rates. Optimization was thus used throughout the entire study, with models sometimes requiring different means of optimization.

In the next section, we begin with the simplest models and examine how they describe the experimental data for the various sequence-rating tasks. Across sections “The Four Initial Models: Peak, End, Peak-End (PE), and Average,” “Relative-Preference-Dependent and Order-Dependent models,” “Absolute-Preference-Dependent Model,” “Windowed Evaluation,” and “Discounting Models,” we found that the experience evaluation model requires (1) more than just averaging; (2) a robustness to scale that predicts sequence ratings for sequences of arbitrary size; (3) a flexibility to capture both local evaluation within a given window of time, as well as more global evaluation of particularly salient experienced events; and (4) model parameters that significantly correlate with preference-dependent working memory.

## The Four Initial Models: Peak, end, Peak-End (PE) and Average

The peak-end rule suggests that the total evaluation is based on the peak and end components of the experience. Therefore, we first analyzed *Peak* and *End* as separate estimators, as well as the *Peak-End* estimator, enabling us to examine how each appears to contribute to the evaluation compared to an estimator based on the *average* of the sequence.

### Model Description

The Peak, End, PE, and Average estimators are written in the forms

(5) $$\begin{array}{l} y_{\text{peak}}\left(t\right)=\underset{1\leq i\leq t}{\max}\{x_{i}\} \end{array}$$

(6) $$\begin{array}{l} y_{\text{end}}\left(t\right)=x_{t}, \end{array}$$

(7) $$\begin{array}{l} y_{\text{PE}}\left(t\right)=\frac{1}{2}\left(y_{\text{peak}}\left(t\right)+y_{\text{end}}\left(t\right)\right) \end{array}$$

(8) $$\begin{array}{l} y_{\text{avg}}\left(t\right)=\frac{1}{t}\underset{1\leq i\leq t}{\sum }x_{i} \end{array}$$

where *x*_*i*_ is the individual sequence-event utility (i.e., each picture's preference rating) of the *i*th position in the sequence and *t* is the total number of events in the sequence. These estimators have no parameters and can be used immediately without any learning algorithms. By comparing these four estimators, particularly PE and Average, we measured how much the peak-end bias contributed to the evaluation of the experience. Correlation tests were used to compare the predictive performance of each estimator.

### Model Results for Sequence-Rating Findings

**Figure 1:**
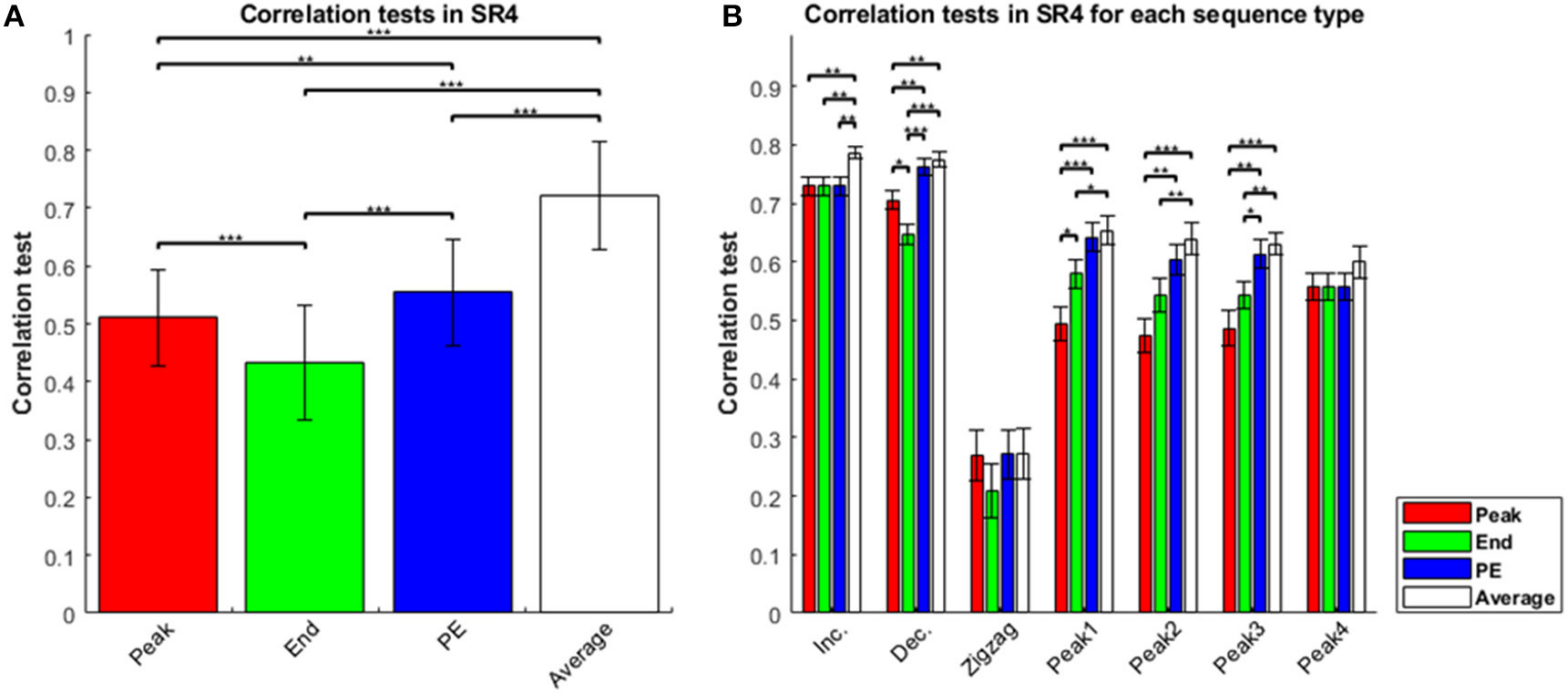
Correlation tests in sequence-rating task with length four. **(A)** Correlation tests for the four sequence-rating estimators. The “Average” had the highest correlation, and the “End” the lowest. **(B)** Correlation tests for the four estimators in seven types of sequences: increasing, decreasing, zigzag, and peak position 1–4 in the sequence. Zigzag sequences were difficult to estimate, suggesting that the estimation occurred at the end of the sequence (rather than making a running estimation throughout the sequence), while the average estimator had the highest correlation for all types. *P*-values of *t*-test: *p < 0.05; **p < 0.01; ***p < 0.001.

We conducted a statistical analysis to compare the four estimators in the sequence-rating task with length four, length seven, and the sequence-rating-continued-version task. In the sequence-rating task with length four, the four estimators correlated with the participant ratings in the order Average > Peak-End > Peak > End (Figure 1A). All comparisons between estimators were significant to at least *p* < 0.01 (for Peak-End > Peak). Analyzing by sequence type—i.e., increasing, decreasing, zigzag, and peak position 1–4 in the sequence—the mean correlation test for Average was always the highest; indeed, the sign test with null hypothesis “all four estimators have the same correlation value” and cases of “Average showing a better correlation test result (*p* < 0.05 in *t*-test) than at least two other estimators of three” as the positive occurrences for the sign test support that the Average estimator was the best of the four, with *p* =(¼)^5^ = 0. 0009766 < 0.001 significance. After Average, the Peak-End estimator was the second best, whereas Peak and End assessment depended on the sequence type (Figure 1B). By definition of the estimators and the sequence types, Peak and End estimators have the same value as the Peak-End estimator in increasing and peak-4 type sequences, because the highest preference appears at the last of the sequence. For increasing and peak-4 type sequences, we thus have only two real estimators, Averaging and Peak-End (with the latter the same as Peak and End). For this reason, peak-4 type sequences show different behavior from the peak-1–3 sequences in Figure 1B.

The results thus consistently show that the Average estimator was the best of the four estimators. Not only was the finding clear with Average being highest in Figure 1, there was further evidence that the estimator closer to the Average showed a better correlation-test result. In decreasing sequences, Peak indicates the first experience and the experiences gradually change from the highest first to the lowest last. Therefore, the Peak-End estimator, which computes (Peak+End)/2, can be understood as an approximation of the average of the sequence. This is why Average and Peak-End did not show a significant difference. Because the Average estimator showed a better correlation, we can say that the Peak-End estimator was a weaker imitation of the Average. In peak-1–3 type sequences, the Peak estimator had lower scores than the other estimators. In the peak-1–3 type sequences, the pictures in the three sequence positions except for the peak had similar preference values, and so the non-peak values were all closer to the average; consequently, the peak value was farther from the average, while the end value was closer to the average. In the peak-4 type sequences, i.e., the peak at the end, the three estimators except Average all collapsed to the same, resulting in the Peak, End, and Peak-End estimators all being farther from the average, again supporting that the estimator closer to the Average performs better.

Even while providing further evidence that Average was the best estimator of the four, the results for the peak-1–3 type sequences provide additional evidence for other effects as well. To see this, imagine setting the peak preference to 1 and all non-peaks to 0, then the Average estimator evaluates as 0.25, End evaluates as 0, Peak evaluates 1, and PE evaluates 0.5. Distance from the Average can be seen as the same for End and PE at 0.25. However, Figure 1B shows that PE had a higher correlation-test value than End. This result cannot be explained if Average is the only true estimator. The result implies that the remembered utilities are not completely explained by Average even with Average being the best of the four estimators, and thus there might be better models than Average alone that also contain some biases toward Peak-End.

**Figure 2:**
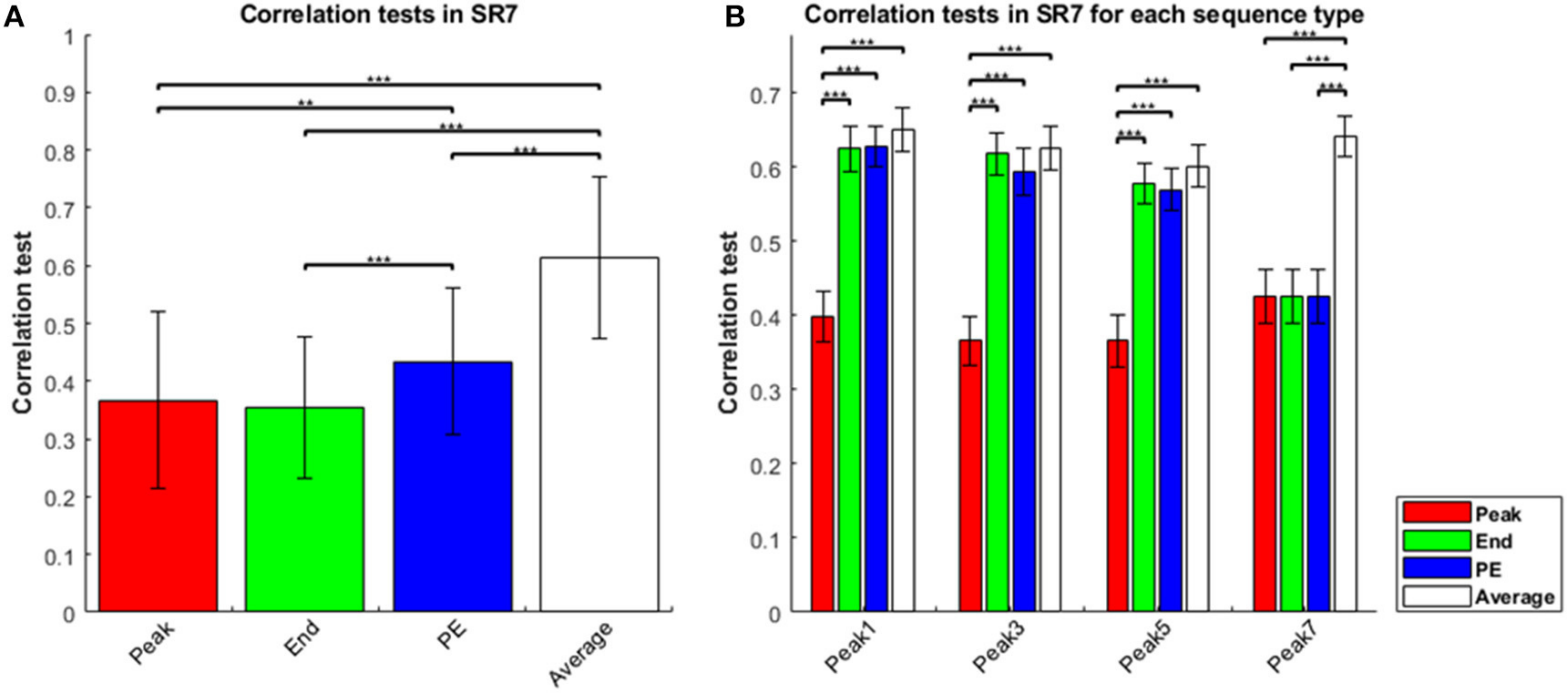
Correlation tests in sequence-rating task with length seven. **(A)** Correlation tests for the four sequence-rating estimators. Again, the average had the highest correlation. **(B)** Correlation tests for the four estimators in the four peak-type sequences. *P*-values of *t*-test: **p < 0.01; ***p < 0.001.

The results of the sequence-rating task with length seven were generally the same as that for length four above. The four estimators again showed Average > Peak-End > End, and Average > Peak-End > Peak (Figure 2A). Thus, averaging of the entire experience always captured the results better. Analyzing by sequence types, we show results for sequence types of peak-1,-3,-5,-7 in Figure 2B. We again find that Average is the best of the four estimators for sequence length seven.

Again examining the specific sequences provides additional evidence for the Average estimator, with the other estimators improving as they approach the average. Because the sequences of length seven are all peak-type, we can compare the correlation tests of length four and seven for peak-type sequences in Figure 1B (peak-1,-2,-3) and Figure 2B (peak-1,-3,-5). For peak-type sequences, the End estimator is closer to the Average than the Peak is, as explained above (i.e., with therefore *end* and other non-peak sequence positions being comparable and lower than *peak*). This similarity becomes greater as the sequence becomes longer (with the irregular peak experience constituting a smaller portion of the longer sequence). Thus, for peak type sequences, End is closer to Average in the length seven case than for length four. Correspondingly, the correlation test of End was significantly smaller than that of Average for peak-1,-2, and−3 type sequences in the length four case, but indistinguishable (*t*-test, *p* ≥ 0.05) to that of Average for peak-1,-3-, and−5 type sequences in the length seven case, as End becomes closer to Average. Moreover, the correlation test of End was significantly greater than that of Peak only for peak 1 type sequences of the three peak types 1, 2, and 3 for the sequence length four case, but was significantly greater than for Peak for all three peak types 1, 3, and 5 for sequence length seven. For some cases (peak 3 and 5) for sequence length seven, the correlation test of End was even higher than the correlation of the Peak-End estimator. Taken together, these results show that the estimator closer to the Average works better, again indicating that Average was the best of the four estimators.

With the result that the Average estimator is the best among the four, two critical issues are raised: (1) whether the sequences are actually being evaluated by the participants retrospectively, or whether the sequence is evaluated and updated along the way; and (2) even if evaluated retrospectively as a sequence, the extent to which these findings scale to longer sequences—that is, whether averaging would still prevail.

Regarding the first issue, whether participants were actually performing retrospective evaluations, the results for the zigzag sequences provide fairly strong evidence that they were. As seen in Figure 1B, the correlations are much smaller for the zigzag sequences than the others (monotonic- and peak-type). Since there should be no extra difficulty evaluating the zigzag patterned sequence picture-by-picture, the results suggest that participants were not in fact evaluating the sequence via a running rating after each experience. The difficulty with zigzag then suggests that the participants were indeed evaluating the sequences at the end, with apparently some need for a pattern in the sequence to assist the evaluation. That is, this difference in the correlation tests of the four estimators between simpler (monotonic, peak types) and more complex patterns (zigzag type) suggests that the participants' underlying cognitive-memory mechanisms actually used the patterns themselves as means to organize, categorize or “chunk” the individual experiences into a sequence. In any event, we can say that the participants were properly viewing and evaluating the four and seven experiences as a sequence.

**Figure 3:**
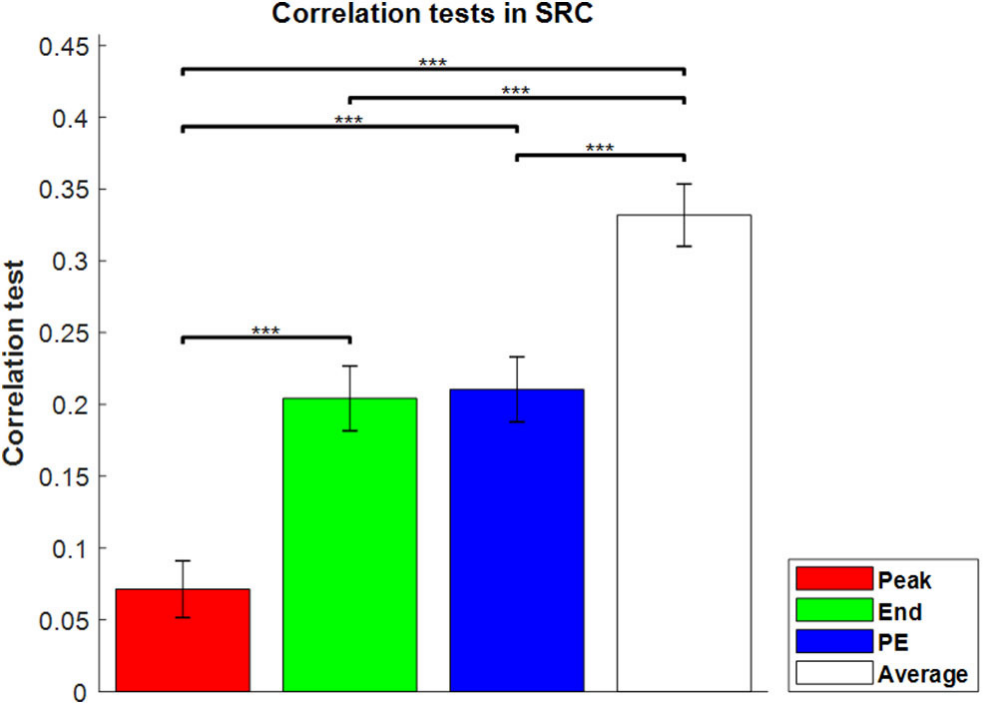
Correlation tests in the sequence-rating-continued-version task. Correlation tests for the four sequence rating estimators. The average again had the highest correlation, and the peak had the lowest. *P*-values of *t*-test: ***p < 0.001.

For the second critical issue, whether sequence lengths four and seven would not scale to longer lengths perhaps more naturally realistic, it is interesting that the results for the sequence-rating-continued-version task were similar to those for sequence length four and seven, which are shown in Figure 3. With the longer sequence, the power of Peak became even much worse and End and Peak-End were indistinguishable with Average > Peak-End = End > Peak order.

In conclusion, averaging all the ratings of the pictures in a sequence provided a better fit to the sequence ratings in all tasks. In addition, the longer the sequence, the stronger the end estimator, and the weaker the peak estimator. From the results broken down by sequence types, the closeness to averaging held a decisive influence on the performance of the estimators.

However, given that all of the estimators (Peak, End, PE, Average) showed significant individual correlations with the participant data (Figure 1A), it suggests that averaging, being the best of the four estimators, might be improved with parametric models that combine its explanatory power with that of the Peak and End estimators. We examine this possibility in the next section, using relative-preference-dependent and order-dependent models as generalizations of the Peak and End estimators.

## Relative-Preference-Dependent and Order-Dependent Models

We studied four basic models related to the peak-end hypothesis in the previous section. Such models based on averaging, picking one specific moment (peak or end), or averaging those specific moments (like PE) are all linear models, which can be expressed as linear combinations of the given sequence. In this section, generalized versions of these linear models are introduced. Specifically, this section aims to find the best linear model to capture the participant results in light of the results from the previous section that multiple components of the sequence appear to influence the sequence ratings.

### Weighting Moment Utilities in the Four Models

To start, Average, Peak, End, and PE estimators, as linear models, are specified by the weights in linear combination:

(9) $$\begin{array}{l} \text{Average}:\;w_{i}=\frac{1}{n} \end{array}$$

(10) $$\begin{array}{l} \text{Peak}:\;w_{i}=\left\{ \begin{array}{cc} 1, & i\text{ indicates peak}\\ 0 & \text{otherwise} \end{array}\right. \end{array}$$

(11) $$\begin{array}{l} \text{End}:\;w_{i}=\left\{ \begin{array}{cc} 1, & i\text{ indicates end}\\ 0, & \text{otherwise} \end{array}\right. \end{array}$$

(12) $$\begin{array}{l} \text{PE}:\;w_{i}=\left\{ \begin{array}{cc} 0.5, & i\text{ indicates peak or end}\\ 0, & \text{otherwise} \end{array}\right. \end{array}$$

Weights for the Average estimator are constant and independent of the sequence elements, indexed as *i*, meaning that there is no assumption on the weights and the weights are unbiased and uniformly distributed.

However, the Peak estimator is dependent on the sequence position, i.e., the index *i*, particularly at the point in the sequence that is most preferred. In other words, the weight is dependent on the position with the highest relative preference. Similarly, the End estimator is dependent on the position index indicating the latest one, i.e., last in the order.

To try to capture all of these effects together in a generalized model, or in fact find even better models than simply combining these four effects (peak, end, peak-end, and averaging), we may simply allow the weights to have other values rather than from the four original models. For example, we can give weights 0.9 for the peak and 0.1 for the second peak to form another model, similar to but different from the Peak estimator, as we consider in the next section.

### Order-Dependent and Relative-Preference-Dependent Models Specified

In this section, we look to take the best current estimator, the Average, and improve upon it. We propose two assumptions on the weights in this section for the generalization of the End and Peak models, respectively, to be combined with the Average estimator. These two assumptions are related to the general problem of how best to index the event sequence in summation—that is, as a weighted sum across the set that best captures the sequence effects.

The first one is an order-dependent assumption *w*_*o*_:

(13) $$\begin{array}{l} w_{i}=\frac{1}{n}+w_{o,i} \end{array}$$

where *w*_*o, i*_ is the weight for the *i*th *position* in the sequence, where *i* = 1 corresponds to the earliest experience, and *i* = *n* corresponds to the latest one. Thus, the weighting is meant to capture not only the average, but any order-dependent effects in the results as well. The second assumption is a relative-preference dependent assumption *w*_*p*_:

(14) $$\begin{array}{l} w_{i}=\frac{1}{n}+w_{p,i} \end{array}$$

where *w*_*p, i*_ is the weight for the *i*th *rank* in terms of relative preference, where *i* = 1 corresponds to the least-preferred experience, and *i* = *n* corresponds to the most-preferred experience. Thus, the weighting is meant to capture not only the average, but any additional relative-preference effects in the results as well (generalizing the Peak estimator to relative preference more broadly). An additional assumption is having zero mean to maintain the sum of the weights to be one.

To determine the weights, we computed those that maximize the correlation tests. Because these are linear models, minimizing the mean squared errors (MSE) guarantees that the correlation is maximized (see section “Experiments, ‘Test for total utility models'” for more detail). We first computed the weights from multivariate linear regression. Then we divided the weights by the sum of the weights, to guarantee that they sum to one, and subtracted by $\frac{1}{n}$ to isolate the effects of the two dependencies over just averaging.

### Results for the Order-Dependent and Relative-Preference-Dependent Linear Models

Minimizing MSE, we found the best-fit models with the order-dependent and relative-preference-dependent assumptions. The results with the order-dependent assumption are shown in Table 1. The weights (second row) are not changed much from unbiased (Average) weights, yet we can see that the sequence is biased to the latest indices and less on the previous sequence events. We can also see a small primacy effect *w*_1_ > *w*_2_. From the result *w*_4_ = 0.2786 > 0.25, it can be concluded that End helps Averaging improve the correlation. However, the fact that averaging dominates experience evaluation does not change because the order-dependent correlation is not statistically distinguishable from that of the Average estimator with *p* = 0.1833.

**Table 1:** Order-dependent best fit linear model, compared to End and Average estimators.

	***w*_1_**	***w*_2_**	***w*_3_**	***w*_4_**	**Corr**.
End	0	0	0	1	0.4331
Ord. dep.	0.2325	0.2224	0.2665	0.2786	0.7424
Average	0.25	0.25	0.25	0.25	0.7216

The results for the relative-preference-dependent assumption are shown in Table 2. The weights (second row) were fit to the relative preference of the images in the sequence from lowest to highest. Again, relative-preference dependence helps averaging improve the correlation, but the Average estimator nonetheless still dominates the evaluation, giving an undistinguishable difference in correlation values between Average and Relative-preference (*p* = 0.1291).

**Table 2:** Relative-preference-dependent best-fit linear model, compared to Peak and Average estimators.

	***w*_1_**	***w*_2_**	***w*_3_**	***w*_4_**	**Corr**.
Peak	0	0	0	1	0.5105
Rel. pref. dep.	0.1781	0.1697	0.2762	0.3760	0.7450
Average	0.25	0.25	0.25	0.25	0.7216

Thus, we found that both order dependence and relative-preference dependence did improve the evaluation model in terms of increasing the correlation value. The two models have correlations of 0.7424 and 0.7450, which are almost the same with *p* = 0.8597. The next issue is whether they are essentially the same estimators, or different estimators capturing different factors with similar prediction performance. We therefore examined the potential differences in their explanatory power for the specific sequence types. We evaluated the Pearson's correlations between total utilities generated from the order-dependent and relative-preference-dependent models, computed by types of sequences (Supplementary Table 1). In peak-type sequences, the predictions of the two models were all correlated above 0.8. However, we found that their correlation was relatively low (0.4305) in the zigzag-type sequences, suggesting that they are in fact distinct factors.

Because we found evidence for both order and preference-dependence effects, we next considered a combined model, which can be understood as a generalization of the PE estimator using both the *w*_*o*_ and *w*_*p*_ assumptions for weights:

(15) $$\begin{array}{l} w_{i}=\frac{1}{n}+\lambda w_{o,i}+\left(1-\lambda \right)w_{p,i} \end{array}$$

where *w*_*o, i*_ and *w*_*p, i*_ are from the previous best fit models, λ = 1 corresponds to the order-dependent best fit model, and λ = 0 corresponds to the relative-preference-dependent best fit model. It would have been best to re-compute all eight weights independently, but the two sets of sequence-event utilities {*x*_*i*_} with different indexes have high autocorrelation so optimization does not work well. Using partially optimized weights as an approximation for the combined model, however, was enough to compare how each assumption contributes.

**Figure 4:**
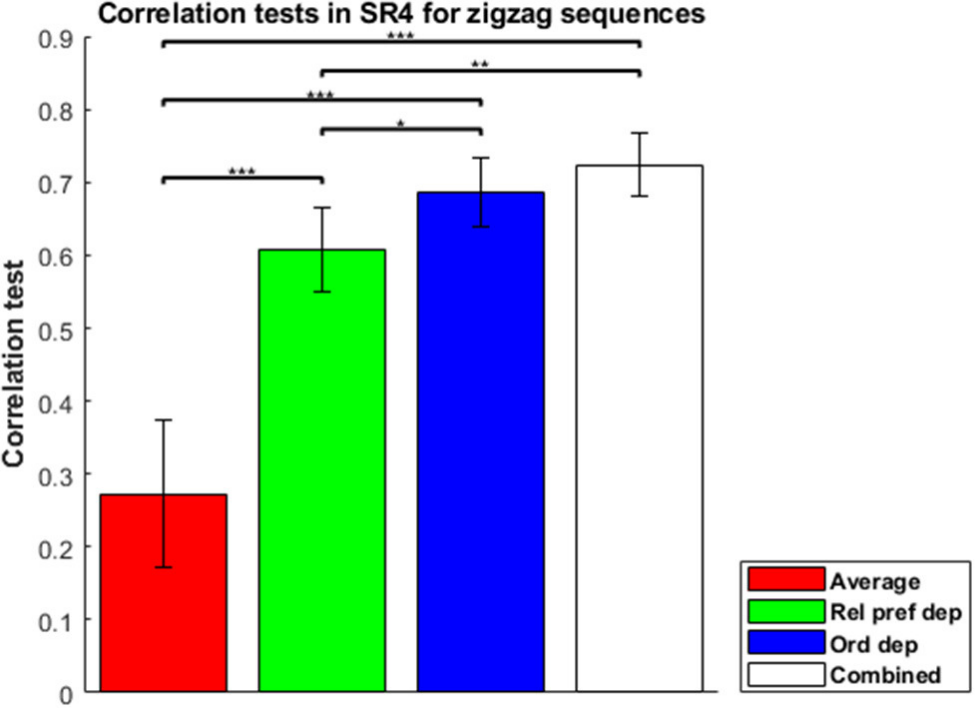
Correlation tests in the sequence-rating task with length four for zigzag sequences. Correlation tests for Average, Relative-preference dependence, Order-dependence, and Combined models. The Combined model produced a 0.7238 correlation. The Order-dependent model produced a better correlation than the Relative-preference-dependent model. *P*-values of *t*-test: *p < 0.05; **p < 0.01; ***p < 0.001.

To evaluate the combined model properly, in which separable contributions of each component (order dependence and relative-preference dependence) are clearly made, we focused on the zigzag sequences, and correlation tests of each model for zigzag sequences are shown in Figure 4. The combined model was the best and Average the worst, because Average is a special case of the others and the combined model is a generalization of the others. The Order-dependent model also showed a higher correlation than the Relative-preference-dependent model. We next examined the composition ratio of the combined model, λ, for all participants (Supplementary Figure 1 histogram). Most participants showed a mixed effect of average, order and relative-preference dependence. At the same time, individual differences were apparent, including multiple participants at the extreme λ = 1 and λ = 0. There also were more participants with λ = 1 than with λ = 0, which explains the correlations in Figure 4—i.e., that the order-dependent model produces a better correlation than the relative-preference-dependent model.

Models in this section are linear models with *n* weights to be determined. We examined sequences with length four, but if sequences become longer, more weight parameters are needed to be determined requiring more trials in the experiment. However, the number of trials we can test decreases as the sequence length increases, because longer sequences lead to greater fatigue in the participants. Our sample number was chosen to maintain participant attention and alertness, which was not enough to further analyze the sequences with length seven and for the continued-version task.

Taken together, then, we found evidence that for most participants their retrospective sequence evaluations appeared to be based on preference *averaging*, the sequence *order*, and an additional *preference-dependence* weighting. With respect to preference dependence, however, in the relative-preference-dependent model, the experiences were indexed so that the most preferred moment, 2nd preferred, and so on have their own weights. However, if the most preferred sequence event has utility equal to 9, for example, and the second preferred event in the sequence has utility 8.99, these two events have almost the same utility but yet are differently weighted based on the relative preferences, i.e., their ranks. Thus, if the actual preference structure were [1 1.01 8.99 9], there would really be only two kinds of preferences, and yet the four events are weighted by [*w*_1_ *w*_2_ *w*_3_ *w*_4_] in the relative-preference-dependent model with *w*_1_ ≠ *w*_2_ and *w*_3_ ≠ *w*_4_. As a second example, if there were no strongly preferred peak, like [1 1 1.5 1], would participants actually weight 1.5 as a peak? In the next section, we therefore examine another type of preference-dependent model that weights absolute preference (rather than relative) to address these issues.

## Absolute-Preference-Dependent Model

To examine preference-dependent effects in the sequence, we sorted the individual sequence event utilities in the previous section and indexed them as the most preferred, second preferred and so on—and thus based on their relative preference. A potential issue here is that such ranking could misrepresent actual preference, if minor preference differences exist across the sequence items, with the relative weighting artificially magnifying the differences, at least for some participants. We therefore next develop a model with weights again based on preference, however, with events in the sequence with the same basic preference now having the same weight.

### Model Description

Here, weights are dependent on the utility of each event in the sequence, which can be understood as an absolute preference:

(16) $$\begin{array}{l} w_{i}=\frac{1}{n}+f\left(x_{i}\right) \end{array}$$

where *x*_*i*_ again is the individual sequence-event utility, and *n* is the total number of sequence events. Note that this is not a linear model because the weight is dependent on the individual sequence-event utility *x*_*i*_ and is multiplied to each event's utility *x*_*i*_ again. There can be an arbitrary dependence by parametrizing the weights w_i_ freely making the model highly generalized and non-linear. The sum of weights is not fixed for a given sequence, so it cannot be controlled to be summed to 1 like linear models.

To compare the absolute-preference-dependent model with the linear models, we analyzed the sequence-rating task with length four. For fair comparisons, we used four parameters as in the order-dependent and relative-preference-dependent models. The range of the individual sequence-event utility values were divided into four partitions. If the minimum was 1 and maximum 9, the range was partitioned to 1–3, 3–5, 5–7, 7–9. The weight for each sequence event was determined by which partition the utility value was included. Weight *w* was *w*_1_ for 1 ≤ *x* < 3, *w*_2_ for 3 ≤ *x* < 5, *w*_3_ for 5 ≤ *x* < 7, and *w*_4_ for 7 ≤ *x* < 9.

### Results for the Absolute-Preference-Dependent Model

The best-fit absolute-preference-dependent model had a mean 0.7430 correlation value with 0.0861 standard deviation, indistinguishable (*p* = 0.9696 and 0.8901 in *t*-test) from the order-dependent (0.7424) and relative-preference-dependent (0.7450) models. To determine if the models were truly indistinguishable, we next examined the correlations between the predictions of the absolute-preference-dependent model and the two linear models (i.e., the order-dependent and relative-preference-dependent models) by sequence types (Supplementary Table 2). The correlations in zigzag sequences were 0.3749 and 0.3093 between the absolute-preference-dependent model and the order-dependent and relative-preference-dependent models, respectively, showing that, although all three models produced virtually indistinguishable correlations with the overall sequence ratings, the absolute-preference-dependent model nonetheless uses different features from the two other models.

**Figure 5:**
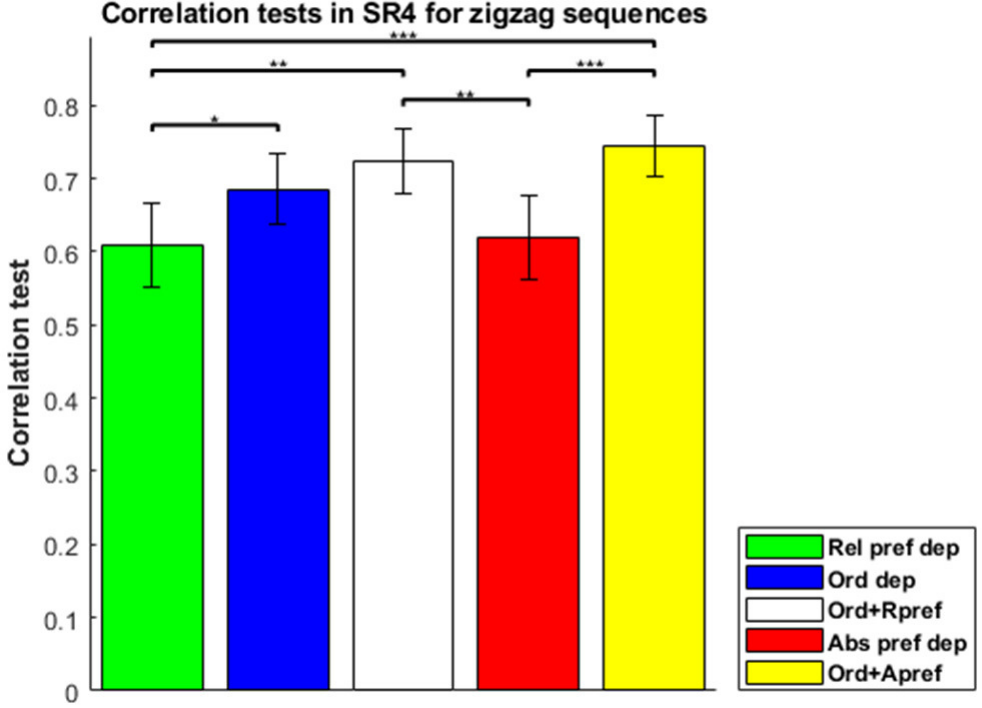
Correlation tests in the sequence-rating task with length four for zigzag sequences. Correlation tests for the Relative-preference-dependent, Order-dependent, Absolute-preference-dependent, and two combined models. The combined model of the Order-dependent and Absolute-preference-dependent models had the largest correlation of 0.7437. Left three bars are the same as those in Figure 4. *P*-values of *t*-test: *p < 0.05; **p < 0.01; ***p < 0.001.

Correlation tests for the order-dependent, relative-preference-dependent, and absolute-preference-dependent models, together with combinations, are shown in Figure 5 for the zigzag type sequences. The combined model of order-dependent and absolute-preference-dependent models (in yellow) had the highest correlation value of 0.7437, although this value was not significantly different from the correlation (0.7238) of the combined model of order-dependence and relative-preference-dependence (in white) (*t*-test, *p* = 0.5058).

The results thus show that both relative and absolute preference dependence (as well as order and average) appear to influence retrospective sequence evaluations. However, there is an important practical difference between relative and absolute preference dependent models. The relative-preference model parameterizes weights such that the number of parameters is the length of the sequence. It thus quickly becomes infeasible as the sequence becomes long (e.g., sequence length 100 requires 100 parameters). In contrast, the absolute-preference model parameterizes weights such that the parameters are defined on the range of the moment utilities, and the number of parameters is thus smaller than that of the relative model. Here, we use 17 levels of preference values, which then is the maximum number of parameters for the absolute model. Even this number is indeed large, but better than for the relative model.

Because of these difficulties for the relative-preference-dependent assumption, we do not use relative-preference dependence in examining the sequence-rating-continued-version task; the term “preference,” then, refers to *absolute preference* for the remainder of the study. The same problem of too many parameters also occurs for order dependence, which is considered in the next section.

**Figure 6:**
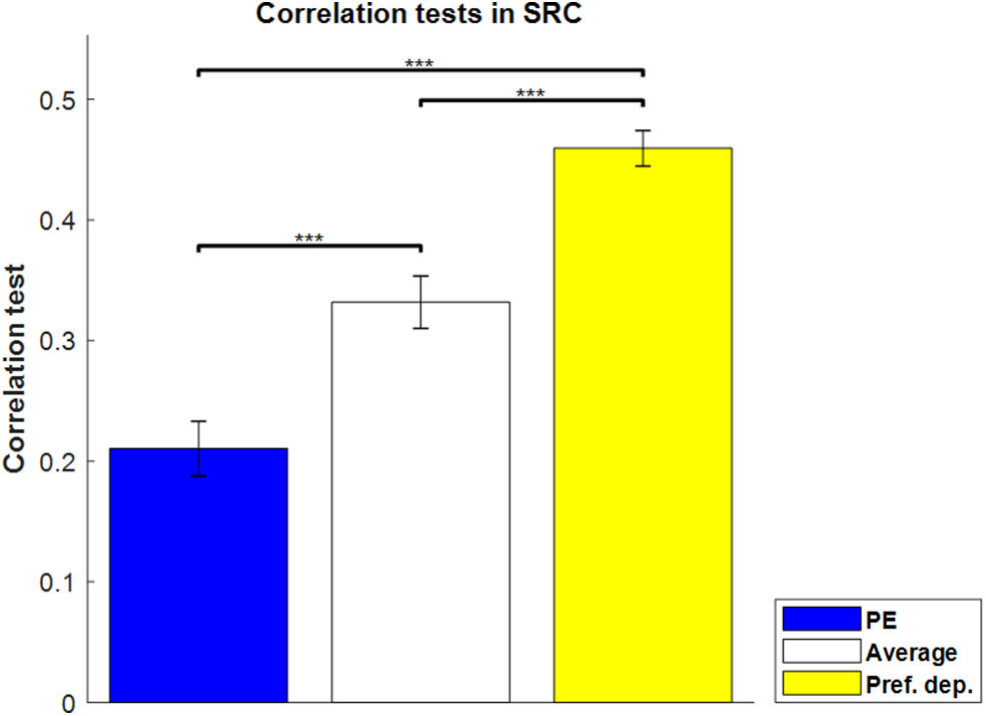
Correlation tests in the sequence-rating-continued-version task. Correlation tests for PE, Average, and Preference-dependent estimators. The Preference-dependent estimator had the highest correlation. Left two bars are the same as those in Figure 3. *P*-values of *t*-test: ***p < 0.001.

For the sequence-rating-continued-version task, then, we can find the best-fit absolute-preference-dependent model, where the length of sequence is not fixed with 100 as maximum. The results are shown in Figure 6. The absolute-preference-dependent model, based on both averaging and absolute-preference (see Equation 16), produces a better correlation than Average alone, indicating again that the actual preferences for the images had an amplified effect on sequence ratings.

### Order and Preference Dependent Models

To this point, we have examined models with weights for each sequence event depending on its temporal order and individual utility (preference), without interaction, potentially still missing important effects. In the remainder of the study, we focus on the effects of temporal order and preference with their interactions included. The general form of the models can be written thus:

(17) $$\begin{array}{l} w=f\left(x,\;t\right) \end{array}$$

To have enough temporal dynamics with long sequences, this model analyzes the sequence-rating-continued-version task. We parameterized preference dependence with linear interpolation of 5 independent variables to determine 17 levels of preferences: [1, 1.5, 2, 2.5, …, 8.5, 9]. For temporal dependence, because the length of sequences was not fixed and with a maximum of 100 in the task, it was hard to parameterize without assumptions. Moreover, our aim was to develop a model with the potential to generalize and scale to every possible length of sequences. Such a model requires an assumption on temporal dependence in a closed form of the equation.

From the results thus far, participants appeared to evaluate the sequences by averaging the individual events within the sequence, with additional influences of order and relative or absolute preference dependence. In the sequence-rating tasks with lengths four and seven it would not seem particularly difficult to memorize 4–7 pictures. However, in the sequence-rating-continued-version, participants would potentially have to memorize up to 100 pictures. This seems highly unlikely; and thus, it does not appear that the participants actually computed averages of 100 pictures. This in turn suggests that the order-dependent assumption should contain working-memory features to handle this memory issue. Therefore, the next two sections use two different temporal assumptions to build more realistic order and preference dependent models. The first assumption simply cuts and reduces the sequence to be evaluated by assuming an optimal temporal window, and then within this, computes and compares the four original models (Peak, End, Peak-End, and Average), along with the preference-dependent model in the reduced sequence. The second model assumes that the weights are temporally discounted, with the discounting ratio preference dependent.

## Windowed Evaluation

In the previous sections, we found that the models that weighted averaging of the entire sequence, along with additional dependencies (order, preference), explain sequence ratings reasonably well. However, these models assume that the participants remember the entire sequence to be evaluated, e.g., up to 100 pictures in the sequence-rating-continued-version task, which would be very difficult to do. Therefore, we next examined more realistic models that limit the calculation of the mean and maximum values to a partial sequence—i.e., an evaluation window—of the sequence. In these types of models, the length of the evaluation window functions as a model parameter. It may also reflect working memory ability: “how many pictures are you using in the evaluation?”

### Model Description

Three suggested new estimators are the following:

(18) $$\begin{array}{l} \text{Windowed PE}:\\ w_{i}=\left\{ \begin{array}{cc} 0.5,\; & i\text{ is peak or end in }t-L+1\leq i\leq t\text{ range}\\ 0,\; & \text{otherwise} \end{array}\right. \end{array}$$

(19) $$\begin{array}{l} \text{Windowed Average}:\\ w_{i}=\left\{ \begin{array}{cc} \frac{1}{L},\; & i\text{ in }t-L+1\leq i\leq t\text{ range}\\ 0,\; & \text{otherwise} \end{array}\right. \end{array}$$

(20) $$\begin{array}{l} \text{Windowed Preference}-dependent:\\ w_{i}=\left\{ \begin{array}{cc} w_{i}\left(x\right),\; & i\text{ in }t-L+1\leq i\leq t\text{ range}\\ 0,\; & \text{otherwise} \end{array}\right. \end{array}$$

These three models are variations of previous estimators, only using the last *L* terms in the sequence. This class of model does not require memorizing the entire sequence. The size of window *L* is set to maximize the correlation-test value:

(21) $$\begin{array}{l} L=\underset{k}{\text{argmax}}\text{ corr }(y_{k},y) \end{array}$$

**Figure 7:**
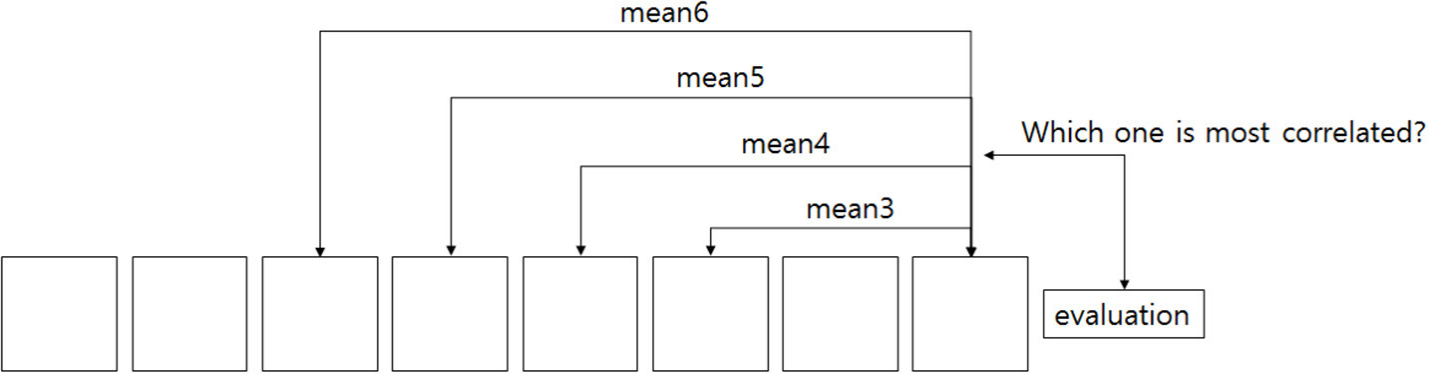
Windowed estimator. Finding the best estimator using only recent pictures. The number of pictures used in estimation becomes the size of the window.

Figure 7 illustrates how we determined the size of the window *L* for the windowed-average estimator. More specifically, the argument of the right term corr(*y*_*k*_, *y*) was computed for all possible window sizes 1 ≤ *k* ≤ 100. When computing the correlation, the samples (i.e., trials) were bootstrapped by removing one element (i.e., trial) from the samples, and correlations were computed from the bootstrapped samples. Bootstrapped correlations (one for each trial removed) were then averaged to obtain the final value corr(*y*_*k*_, *y*). Because the preference-dependent model requires linear regression, we skipped the computation when the number of samples was <30. The maximum tested value of *k* by this process was 49. For fair comparison, the Average and Peak-End models also skipped the same cases. If the bootstrapped mean correlations were under the threshold correlation to achieve *p* < 0.05 significance, the value was not used in the argmax function. To find the maximum value (i.e., of the bootstrapped correlations), we chose the correlation that had the most number of other correlations significantly (*p* < 0.05) less than it.

### Results for the Windowed-Evaluation Models

**Figure 8:**
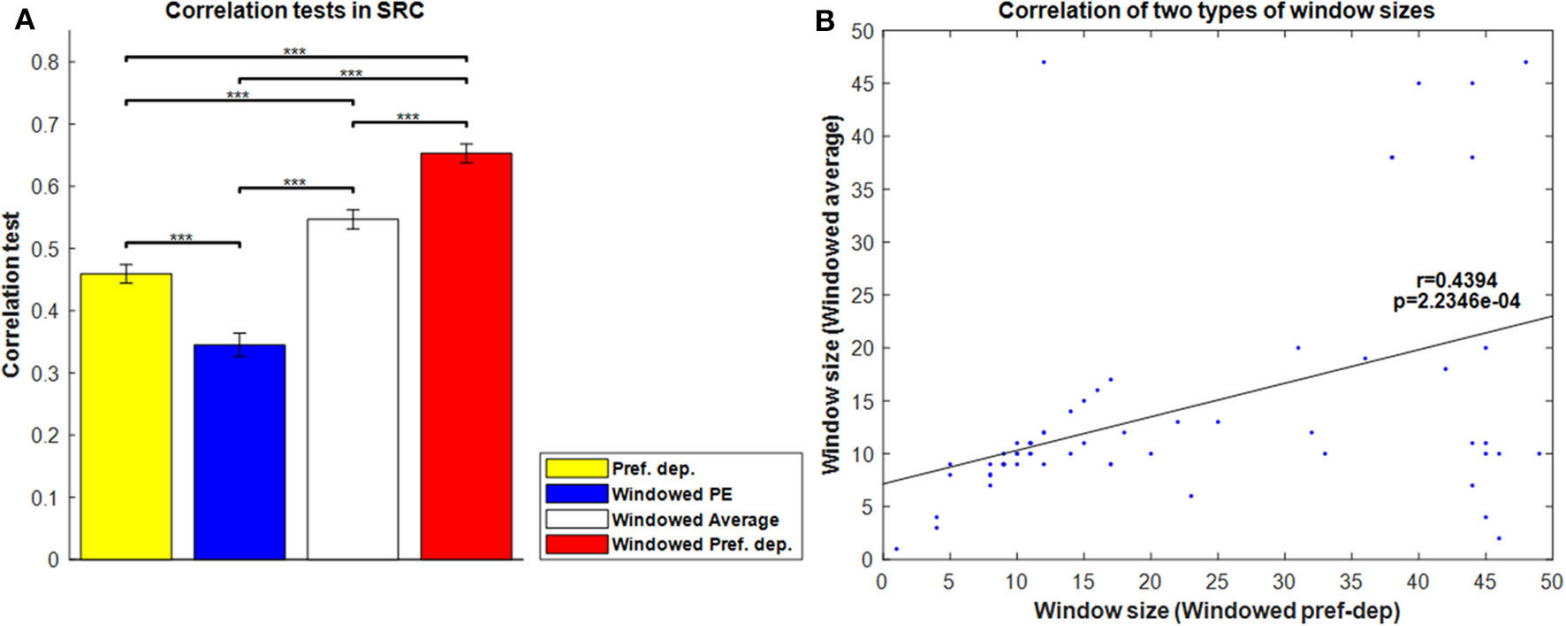
Results on Windowed models. **(A)** Correlation tests for the sequence-rating-continued-version task with Windowed estimators. For long sequences with length up to 100, the Windowed Preference-dependent estimator produced a 0.6529 correlation, while only 0.4594 was achieved without windowing. *P*-values of *t*-test: ***p < 0.001. **(B)** Plot of the window sizes of the Windowed Average and Preference-dependent estimators. The correlation between them was 0.4394 with *p*-value 2.2346e-04.

Figure 8A shows the correlation tests for the Windowed Peak-End, Windowed Average, and Windowed Preference-dependent models, along with the not-windowed Preference-dependent model from the previous section for comparison. The Windowed Preference-dependent model produced a correlation of 0.6529, the best correlation test of the four models in Figure 8A, with significant differences compared to the other three models in *t*-test. Moreover, the correlation test of the Windowed Preference-dependent model was close to the correlation test of the preference-dependent model in the sequence-rating task with length four (0.6201, see Figure 5), suggesting that the windowed model can explain the remembered utilities for long sequences as well as short sequences, which was not feasible for not-windowed models.

We next compared the two window sizes derived from the Windowed Average and Windowed Preference-dependent models. Figure 8B shows the correlation between the two window sizes. They are highly correlated with correlation 0.4394 and *p*-value of 2.2346e-04. This similarity of the derived window sizes suggests that there is indeed a meaningful evaluation window for each participant, independent from how it is estimated (average or preference-dependent). At the same time, however, the window size for the Windowed Average estimator clustered at 10, the mean period of participant evaluations in the task (i.e., how often the evaluation of the current running sequence was queried), while the window size for the Windowed preference-dependent model did not. This difference indicates that the window size for the Preference-dependent model is relatively free from the period of evaluation and can have large values while that of Averaging is locked with the period.

The result in Figure 8B provides clues about the memory mechanisms underlying the two windowed models. Window sizes for Windowed Average are clustered at the period of evaluation: 10. It implies that there is a memory mechanism that deals with a piece of memory composed of one period of evaluation that the Windowed Average model captures. However, window sizes for the Windowed Preference-dependent model can be larger than the period of evaluation and spread out. This suggests that the Windowed Preference-dependent model is not restricted to local memory of one period and that participants can use a global memory composed of more than one period of evaluation. The model results together suggest that the participants are not evaluating only for any one particular period, but for multiple periods in an attempt to cover the entire sequence in our SRC task paradigm. In other words, the difference in distribution of window sizes for the two models implies that there are two general levels of memory—a local memory of one period and a global memory of multiple periods—and that the Windowed Preference-dependent model works on a higher memory level than Windowed Average. We will not deal further with this potentially fascinating suggested finding, as our experiment was not designed to address it (not allowing us to model it further), though we will return to it briefly in the Discussion.

In this section, we investigated how windowed models fit the empirical data, and found that the Windowed Preference-dependent model successfully explained the results of the sequence-rating-continued-version task. Nonetheless, windowed models have some inherent limitations, most notably three:

- Primacy effect problem. A primacy effect in experience evaluation for some cases has been well-established; and it is not hard to imagine that one gets a strong impression from the first picture of a sequence. Windowed evaluation cannot readily account for the primacy effect because it is not concerned with the first experience in evaluation, which is often out of the evaluation window.
- Extreme peak problem. If one has a particularly meaningful or intensive experience anywhere in the sequence, it would be expected to affect the experience evaluation, regardless of the sequence length. But if that experience is out of the evaluation window, it is entirely neglected.
- Boundary cliff problem. In windowed evaluation, an experience just before the window border has no effect at all, while an experience just after the boundary has full effect. It is hard to accept that the two experiences of the neighbors have such extremely different effects.

The class of models in the next section was introduced to resolve these problems. Inspired by problem 3 (boundary cliff), we attempted to smooth the window boundary, using a continuous function on the entire sequence as the function domain, leading to a discounting model. To address problem 2 (extreme peak), we added preference dependency in the discounting rate. Finally, given a sufficiently flexible discounting model, primacy effects can also potentially be captured, as described next.

## Discounting Models

To deal with the three main problems (primacy effect, extreme peak, boundary cliff) raised at the end of the previous section for windowed evaluation, as well as capture order effects more generally, we introduce discounting models in this section. Discounting is a common way to describe the limitations of memory, and thus forgetting, multiplying a discount factor step by step in time to capture the loss of the effect of events as they pass farther into the past. In addition, because our results suggest significant preference-dependent effects in experience evaluation, we added preference dependency to the discounting rate of a second discounting model.

With discounting, the weighting for the specific moments in the sequence evaluation is a continuous function, and so there is no boundary-cliff problem. In addition, if the discounting rate is sufficiently high for any given highly preferred experience, an extreme peak can affect evaluation with non-zero weight. Finally, if the discounting rates for early pictures are also sufficiently high, a primacy effect can be captured.

We next introduce specific discounting models with and without preference dependency in the discounting rate and examine their ability to account for the empirical data.

### Model Description

The simple discounting (SD) model and the preference-dependent discounting (PD) model are defined in these forms:

(22) $$\begin{array}{l} y_{\text{SD}}\left(t\right)=\underset{1\leq k\leq t}{\sum }r^{t-k}x_{k} \end{array}$$

(23) $$\begin{array}{l} y_{\text{PD}}\left(t\right)=\underset{1\leq k\leq t}{\sum }{r\left(x_{k}\right)}^{t-k}x_{k} \end{array}$$

where *r* is the rate of forgetting, which connects to the concept of working memory; *t* is the total sequence length; *k* is the position in the sequence; and *x*_*k*_ is again the utility (i.e., preference rating) of sequence event *k*. The difference between the SD and PD models is that the *r* value of the PD model depends on the corresponding *x* value, while the *r* value of the SD model is constant.

The SD model becomes the Average estimator when *r* = 1, and the End estimator when *r* = 0. And since this model reduces the weight of *x* as the experience moves into the past, windowed average can also be simulated. However, the peak and windowed peak cannot be explained because the peaks in the past may have higher weights than the weights of more recent events in the Peak and Windowed peak models, whereas this cannot occur in the SD model whose weights are a decreasing function of time into the past.

**Figure 9:**
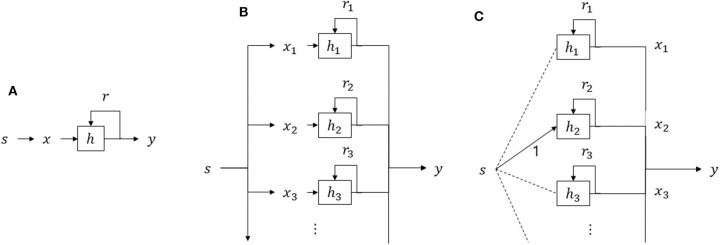
Block diagrams of the discounting models. **(A)** Depicts the simple discounting model. The model transforms the stimulus into the moment utility *x* and the moment utility is then added to the discounting node *h*, which has negative feedback (discounting) *r* to generate total utility *y*. **(B,C)** Show two ways of depicting the preference-dependent discounting model. Compared to **(A)**, the PD model has multiple nodes in **(B)** to produce different discounting effects for different preference levels. In the equivalent diagram **(C)**, the need for stimulus classification by their preference is removed and the preference can be defined as a summation weight at the last layer, as a result of the variety in weights of multiple nodes, described in more detail in the text.

The PD model is a generalization that completely encompasses the SD model, and for some values of *r* and *x*, the weights may in fact increase as they move into the past. Thus, if the preference of a picture viewed in the past is high and the corresponding *r* value is large enough, the peak can contribute to the evaluation even if it is not a recent experience. Block diagrams of the models are shown in Figure 9.

To simplify the block diagram in Figure 9B for the PD model, the equation can be translated in another way, as shown in Figure 9C:

(24) $$\begin{array}{l} y_{\text{PD}}\left(t\right)=\underset{1\leq k\leq t}{\sum }{r\left(x_{k}\right)}^{t-k}x_{k}=\underset{x:\text{preference}}{\sum }\left[x•\underset{1\leq k\leq t,x_{k}=x}{\sum }{r\left(x\right)}^{t-k}\right] \end{array}$$

Equation 24 is a form of recurrent network with recurrent hidden state *h*:

(25) $$\begin{array}{l} h_{i,t}=r_{i}h_{i,t-1}+g_{i,t}\; \end{array}$$

(26) $$\begin{array}{l} y_{t}=\sum x_{i}h_{i,t} \end{array}$$

where *x*_*i*_ is a preference index for node *i* and *g*_*i, t*_ is 0 or 1 activation from the visual perception layer. In this case, the SD model is a special case with only one recurrent node. The block diagram has three layers. The first layer can be understood as a winner-take-all network. It takes a visual stimulus and performs unsupervised classification (clustering) to activate one respective unit, corresponding to a preference level. At the second layer, each node has its own discounting rate to be discounted by time. The third layer is a weighted sum from signals generated by the second layer. In this explanation, it does not require preference information at the first layer. Preference is determined at the third layer (Equation 26) as the weight of summation *x*_*i*_.

This interpretation can explain the origin of preference dependency. Without any assumptions on preference, this model can assign preferences by itself. The node in the second layer with the highest summation weight to the third layer is the node with the highest preference. Pictures classified to this node are the most preferred pictures. The summation weights have natural variances, and they generate the preference levels. If the total utility generated by the recurrent network without preference information is evolved to be correlated with the experiential reward (for survival, mating, quality of life, or anything), then it naturally occurs that the summation weights in the third layer (Equation 26) encode preference levels and the first layer classifies the stimulus by its helpfulness, which the individual feels as a preference. In this study, the first layer is fixed to classify the pictures by its preference and the total utility is proportional to the remembered utility to learn the second and third layers.

### Algorithm

To find model parameters *r*(*x*), a gradient descent algorithm was used. To maximize the correlation test of the discounting models, we minimized the sum of squared errors of the re-scaled predictions, which is mathematically equivalent as described in section “Models, ‘Test for total utility models'”. The learning rate was 0.0001. Using SSE rather than Pearson's correlation, two more parameters for scaling were needed:

(27) $$\begin{array}{l} y_{PD,t}=a•\left[\underset{1\leq k\leq t}{\sum }{r\left(x_{k}\right)}^{t-k}x_{k}\right]+b \end{array}$$

Because a large parameter set can cause overfitting, only a few parameters were set to be free and the others were dependent parameters. The *a* and *b* values could be determined by a simple linear regression formula. Only five *r*-values were free and the others were determined by linear interpolation of the free parameters. These computations are illustrated in the flow chart in Supplementary Figure 2.

### Results With Correlation Test

To evaluate the two discounting models, we computed correlations as in the previous sections. Because we maximized the correlation test (by minimizing SSE), and the SD model is a special case of the PD model, the correlation test of the PD model must be higher than the correlation test of the SD model if gradient-descent optimization was successful.

Simulation results using the sequence-rating-continued-version task data showed that the correlation of the PD model was significantly higher than for the SD model (Supplementary Figure 3), which means that the PD model's explanatory power is better than the SD's in correlation. The result confirmed that GD optimization worked well (by the PD model giving better prediction), and the correlation test of PD was 0.5269 ± 0.0180 (mean ± standard error).

The result that the PD model predicts better than the SD model is not surprising because the PD model has many more parameters and the SD model is a special case of the PD model. What we need to determine is whether the PD model actually used additional degrees of freedom for the implementation of the working-memory ability as described in the model description. Put differently, it is important to test whether the discounting rate is actually related to working-memory ability, which we next did by examining the correlation of preference dependence in the working-memory task to the preference dependence modeled by the discounting rate. To measure preference dependence in the working-memory task, we first calculated each participant's preference-dependent accuracy score as a vector of length four. For example, [0.9, 0.8, 0.6, 0.7] would mean that the accuracy rate was 0.9 for preference group star 1, 0.8 for preference group star 2, 0.6 for preference group star 3, and 0.7 for preference group star 4. We then computed the variance of this vector (preference-dependent working-memory accuracy rate) to measure how their working-memory accuracy was affected by preference. If the preference-dependent working-memory accuracy rate were [0.5 0.5 0.5 0.5] or [0.9 0.9 0.9 0.9], for example, then the variance would be exactly zero and their working-memory accuracy would not depend on preference. If the variance were high, working-memory accuracy would highly depend on preference.

**Figure 10:**
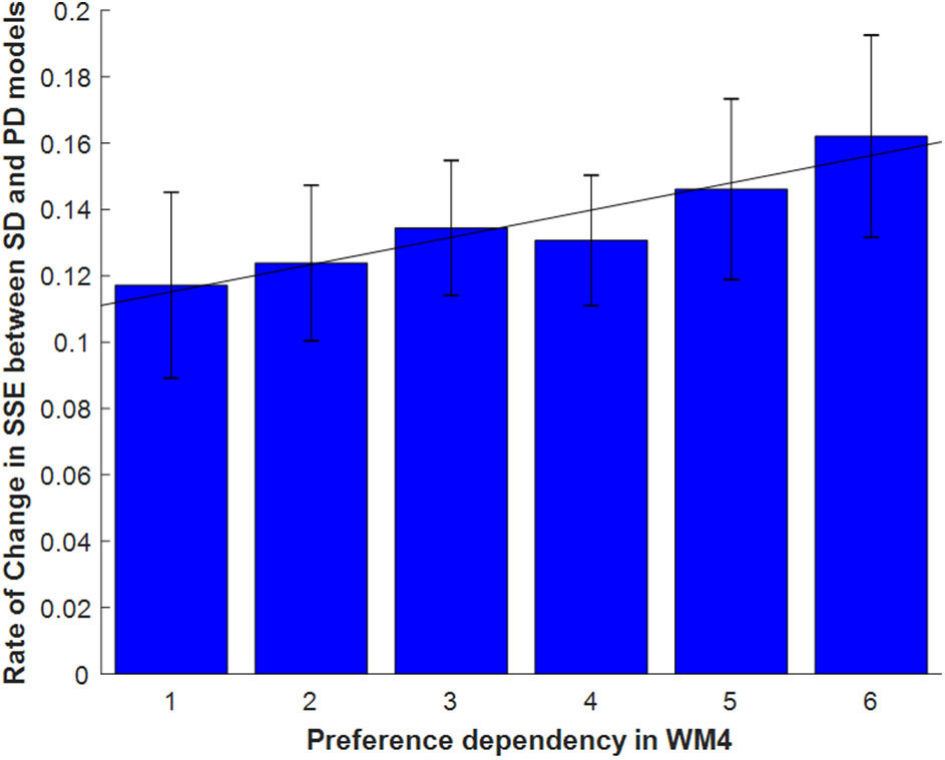
SSE changes from the SD to the PD model in six groups with different degrees of preference dependency in the working-memory task. For x-axis, preference dependency in the working-memory task was measured (see main text), and participants were divided into six groups based on their relative preference dependency from low to high. The y-axis then measures the extent to which the PD model captures this preference dependency, using | [(SSE of PD model) - (SSE of SD model)]/(SSE of SD model) |. Participants whose working-memory performance reflected a low degree of preference dependence (Group 1) showed little performance difference between the PD and SD models, while participants whose working-memory performance reflected a high degree of preference dependence (Group 6) required the PD rather than the SD model to describe their remembered utility. The group index (1–6) was strongly correlated with the improvement from the SD to PD models (*r* = 0.9478, *p* = 0.0040), as illustrated by the fitting line. The result shows that the additional degree of freedom of the PD model was necessary to capture preference-dependent working-memory effects.

Based on the variance, six groups were created ranging from low to high variance, and thus with preference dependence from low to high. If the PD model actually reflected working-memory ability in relation to preference dependence, as the group number increases, it should show better explanatory power over the SD model. Figure 10 shows this relationship. The y-axis of the graph is the absolute value of the rate of change of the SSE of the PD model from the SD model: i.e., | [(SSE of PD model) - (SSE of SD model)]/(SSE of SD model) |. As the group number increased, the explanatory power of the PD model was superior to that of the SD model, meaning that the higher the dependence of working memory on preference, the better the performance of the PD model compared to the SD model. Indeed, group number (1–6), representing the preference-dependency in the working-memory accuracy rate in sequences with length four, was highly correlated with the rate of change in SSE (i.e., the improvement from the SD model to PD model) (*r* = 0.9478, *p* = 0.0040; fitting line in Figure 10). This strong correlation, then, shows that the preference-dependent discounting rate in the PD model successfully captures the preference-dependent working-memory ability.

In sum, the results in this section show that (1) the PD model can explain the remembered utility in retrospective evaluation, and (2) the preference dependence in the PD model captures the preference-dependent working-memory ability. Thus, preference dependence is not a redundant assumption possibly overfitting with the use of the PD model: that is, the improvement from SD to PD is not mathematically trivial, with the preference-dependency assumption actually reflecting preference-dependent working memory of the participants.

In this section we focused on the preference-dependence assumption of the PD model, and we now turn to the discounting rates in the PD model to determine if they capture other non-obvious working-memory features.

### Discounting Rates and Working Memory

In the previous section, we found that the preference dependency is not a redundant assumption in the PD model as it explains the preference dependency found in the working-memory performance of the participants, which could not be captured by the simpler SD model. At the same time, the discounting rates were introduced to capture working-memory effects more generally, with the discounting rates being determined by maximizing the correlation test, using the empirical data of the sequence-rating tasks. Yet if the discounting rates themselves also reflected working memory, we would expect them also to be correlated with the working-memory ability measured in the working-memory task, which we therefore investigated in this section.

We again used the preference-dependent accuracy rates from the working-memory tasks with length four and seven, yielding 4- or 7-dim vectors defined for each participant. For example, the 4-dim vector [0.9, 0.8, 0.6, 0.7] would mean that the accuracy rate was 0.9 for the highest preference category (star 1), 0.8 for the second (star 2), and so on. We then used multivariate linear regression with PCA for the preference-dependent accuracy rates:

(28) $$\begin{array}{l} r=\underset{1\leq i\leq D}{\sum }a_{i}w_{i}+b+\epsilon \end{array}$$

where *D* is sequence length (4 or 7), and *w*_*i*_ is the accuracy rate for preference group *i* of the image being queried (i.e., “How many pictures had you seen before this picture?”). Before regression, we used PCA on the vectors {*w*_*i*_}, and used components that give the most significant regression (F-statistic).

The SD model yields one *r*-value, and the PD model five *r*-values. A multivariate regression analysis was performed to see how well these *r*-values could be explained by preference-dependent working memory (Supplementary Tables 3, 4). The working-memory task with sequence length seven did not provide a significant explanation for the discounting rates, with only one discount rate, r17 of the PD model, significantly explained (*p* = 0.0329). However, in the working-memory task with sequence length four, preference-dependent working-memory performance could explain r1, r5, and r9 of the PD model, even with a “simultaneous” *t*-test, meaning the sum of the *p*-values being under the 0.05 threshold. Thus, three of the five discounting rates for the PD model were correlated with the preference-dependent accuracy rates in the working-memory task with length four. This finding indicates that there was a correlation between the discounting rates and preference-dependent working-memory ability. As in the previous section, we again found that the preference-dependent discounting rates directly link to preference-dependent working-memory performance, suggesting that one's working-memory ability contributes to the discount rate of preference.

### In Combination With the Windowed Preference-Dependent Model

Finally, because we found the Preference-dependent discounting (PD) model and the Windowed preference-dependent (WP) model to be the best performing and most realistic models, we consider them together here to determine the extent they may explain the same underlying phenomena, and whether there is value in their combination.

**Figure 11:**
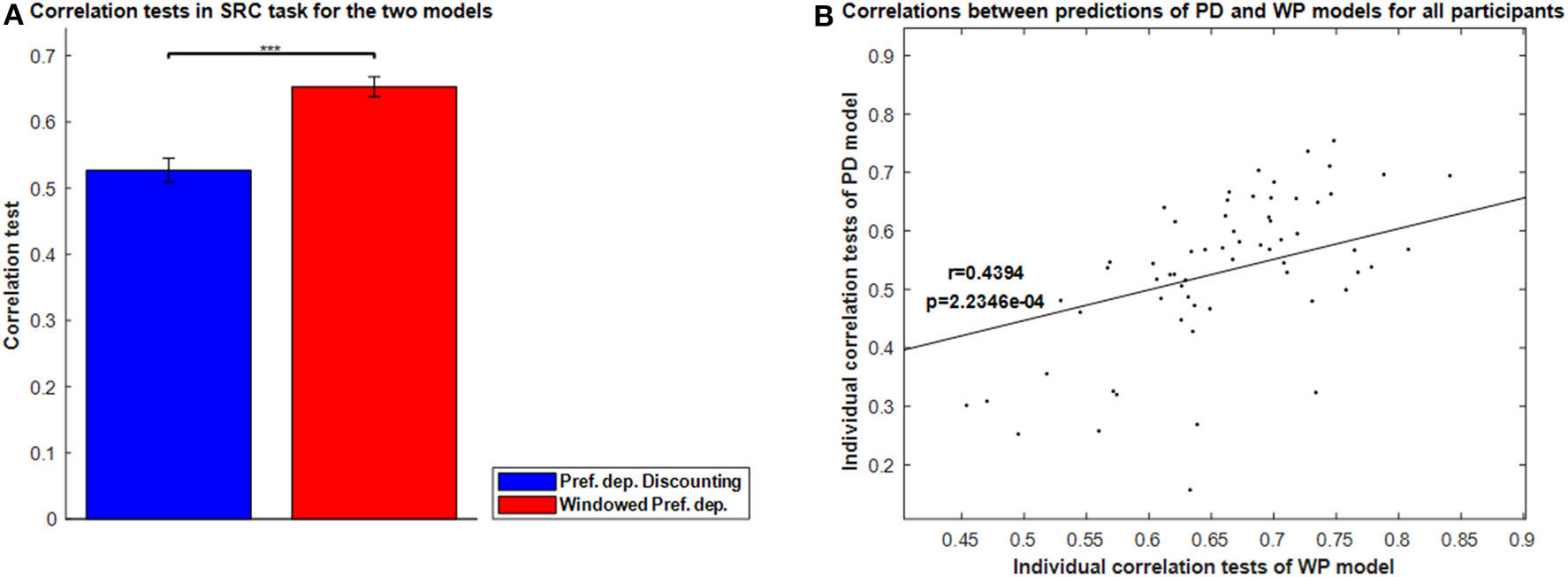
Comparison of the Preference-dependent Discounting model and the Windowed Preference-dependent model. **(A)** The correlation test of the Windowed Preference-dependent model yielded a significantly higher correlation than that of the Preference-dependent model. **(B)** Correlation tests of the two models for each participant are themselves correlated with *r* = 0.4381, *p* = 2.3455e-04, suggesting that the two models describe the empirical data using different factors. *P*-values of *t*-test: ***p < 0.001.

First, Figure 11A directly compares the correlation-test results of the two models. The correlation test of the PD model was significantly smaller than the correlation of the Windowed Preference-dependent model (0.5269 vs. 0.6529, *p* = 3.3292e-07), suggesting either that the Windowed Preference-dependent model performed better than the Preference-dependent model in capturing the same phenomena or that the models each capture different factors, with the Windowed Preference-dependent model focusing on the more dominant factors. To test this, Figure 11B shows that for all 66 participants, the PD and WP models were correlated with *r* = 0.4381, *p* = 2.3455e-04. Even though there appears to be significant overlap between the two models, this level correlation nonetheless suggests that the two models describe the empirical data in different ways. Indeed, as we discussed in the beginning of section “Discounting Models,” the PD model was considered in part because it could account for major weaknesses inherent in the window-based models. This suggests that perhaps the two models could be combined to generate the best model in combination.

**Figure 12:**
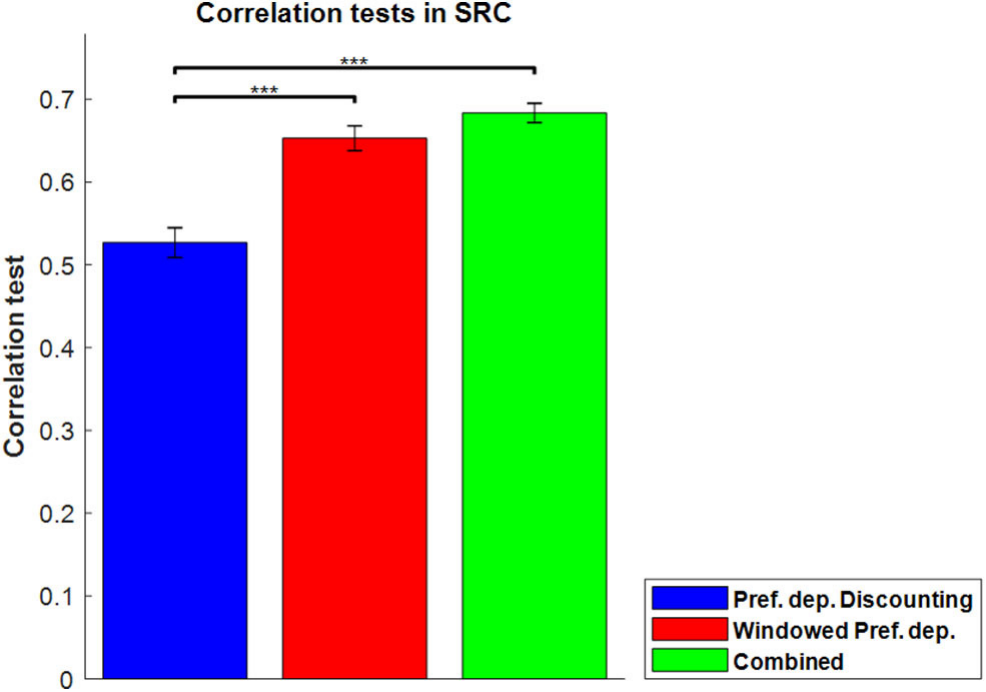
Correlation-tests of the Preference-dependent Discounting model, Windowed Preference-dependent model, and combined WP-PD model. The combined WP-PD model yields the highest correlation, the Windowed Preference-dependent model the second highest, and the Preference-dependent discounting model the third, with the latter being significantly smaller than the other two in *t*-test. ***p < 0.001.

The combined WP-PD model (Equation 29) can be described as a linear combination of the Windowed Preference-dependent model and the Preference-dependent discounting model:

(29) $$\begin{array}{l} y=\lambda y_{\text{WP}}+\left(1-\lambda \right)y_{\text{PD}} \end{array}$$

where λ = 1 corresponds to the WP model and λ = 0 corresponds to the PD model. Correlation tests comparing the combined WP-PD model with the individual models are shown in Figure 12. The combined WP-PD model yielded the highest correlation, the Windowed Preference-dependent model the second highest, and the Preference-dependent discounting model the third, with the latter being significantly smaller than the other two.

**Figure 13:**
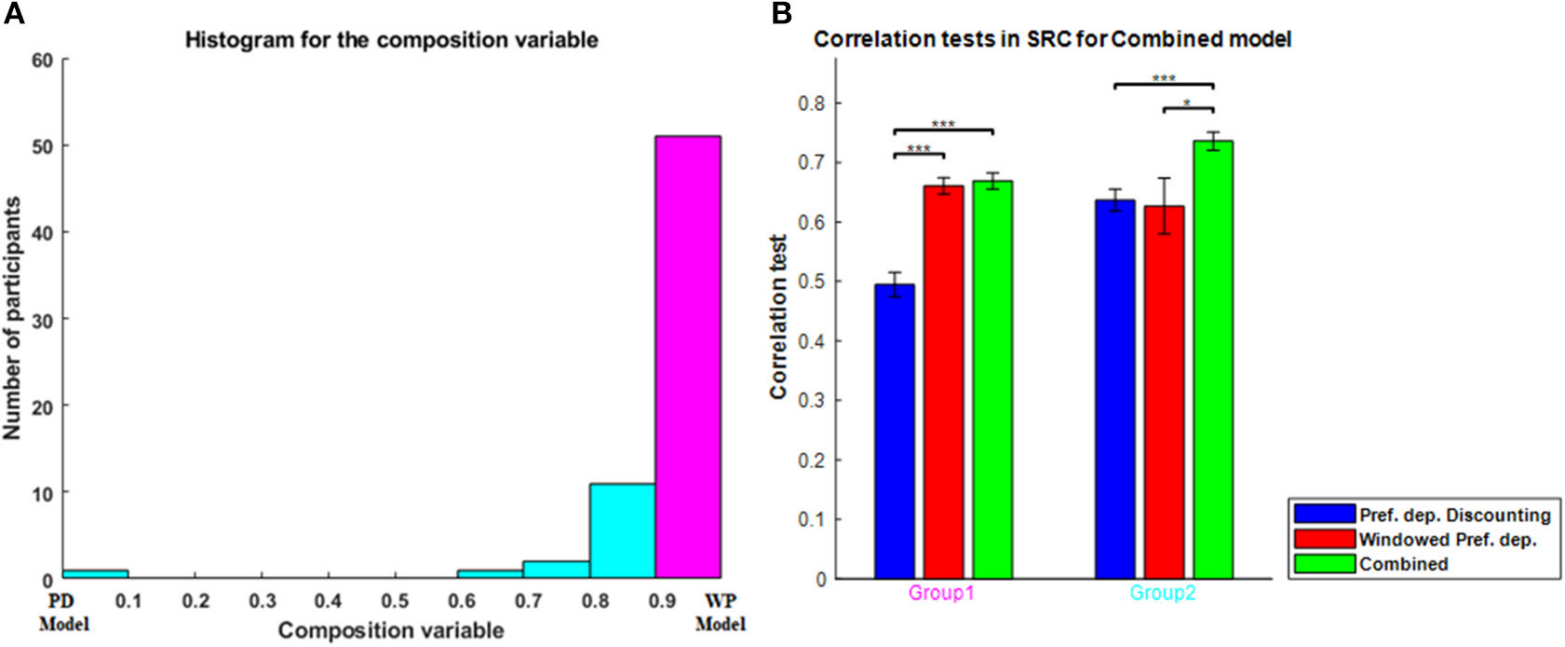
**(A)** Histogram of the ratio of the WP and PD models in the combined WP-PD model, representing the degree to which each model contributes to each participant's evaluations. Participants were divided into two groups with 0.9 cutline. **(B)** Correlation tests of the PD, WP, and combined WP-PD models for the two groups. Participants who clearly used both models (Group 2) showed significant improvements in the correlation test of the combined WP-PD model over the other two models. Fifty-one participants were classified in Group 1 and 15 participants in Group 2. *P*-values of *t*-test: *p < 0.05; ***p < 0.001.

Because the WP model yielded a significantly better correlation-test than the PD model, λ is distributed near λ = 1 (Figure 13A), meaning that most participants were dominantly influenced by the Windowed Preference-dependent factors in evaluation. At the same time, it is clear that the participants could be divided into two groups. Group 1 is classified by λ > 0.9 with participants who mostly utilized Windowed preference-dependent model in evaluation. Group 2 is classified by λ ≤ 0.9 with participants who more strongly used both Windowed Preference-dependent and Preference-dependent discounting factors. Finally, correlation tests of the PD, WP, and combined WP-PD models for the two groups are shown in Figure 13B. For participants who clearly used both models (Group 2), the combined WP-PD model significantly improved the correlation test over the other two models. For participants who predominantly used Windowed Preference-dependent factors, the combined WP-PD model improved the correlation test very little.

In sum, 22.7% of the participants (Group 2) utilized an evaluation pattern that can best be explained by a combination of both the Windowed preference-dependent model and the Preference-dependent discounting model. The other 77.3% of the participants (Group 1) showed an evaluation pattern dominated by the Windowed preference-dependent model. Thus, the combined WP-PD model was able to capture the evaluation patterns of all the participants.

### Examining the Robustness of Pearson's Correlation to Evaluate the Models

Pearson's correlation was used as the main criterion for evaluating the models through the study. To examine how robust this criterion was, we also computed Spearman and Kendall correlations to evaluate the models. The results showed that the type of correlation does not change the conclusions. For example, for the WP model, which gave a Pearson's correlation of 0.6529 ± 0.1221, the Spearman correlation was 0.6428 ± 0.1196 and the Kendall correlation was 0.5124 ± 0.1058. For the PD model, whose Pearson's correlation was 0.5269 ± 0.1459, the Spearman correlation was 0.5160 ± 0.1442 and the Kendall 0.3992 ± 0.1159. Thus, the different correlation measures do not change the fact that WP model predicts better than the PD model on average. Moreover, the combined WP-PD model predicted the remembered utility with correlations of Pearson 0.7359 ± 0.0574, Spearman 0.7238 ± 0.0707, and Kendall 0.5858 ± 0.0657 for Group 2. Thus, again the other types of correlations also indicate that the combined WP-PD model performs better than the WP model alone, as also found with the Pearson's correlation. These comparisons show that the models were robust and not dependent on the measure used to evaluate them.

## Discussion

The organization of singular moments into larger event sequences constitutes a fundamental human high-level cognitive ability (Jeong et al., 2014; Jung et al., 2014; Jang et al., 2017), yet how the human brain actually constructs these sequences and remembers them remains unclear. Evidence suggests that heuristics are used to remember key moments of the sequence, such as the peak-end rule, though findings are mixed (Langer et al., 2005; Rode et al., 2007; Do et al., 2008; Kemp et al., 2008; Liersch and McKenzie, 2009; Legg and Sweeny, 2013; Xiaowei et al., 2013). Therefore, the aims of the current study were to develop an evaluation model to help identify the mechanisms underlying sequence evaluations, and to determine how working memory is involved in the evaluations. To reach these aims, we designed experiments to enable a comprehensive quantitative examination of experience evaluation across different conditions, and to measure the working memory involved in the experience evaluation.

Given that the peak-end rule has received significant attention with supporting evidence (Langer et al., 2005; Do et al., 2008; Liersch and McKenzie, 2009; Legg and Sweeny, 2013; Xiaowei et al., 2013) as a heuristic for retrospective evaluation, we tested it here (along with peak and end individually, and general averaging of all sequence-event preferences). However, our evidence strongly pointed to averaging as the dominant evaluation strategy. At the same time, all initial models (representing peak, end, peak-end, and averaging) nonetheless significantly correlated with the empirical data, suggesting that, although averaging being most prominent, aspects of each may have influenced the retrospective evaluations.

Inspired by the success of the models we sought to develop and test a more general one that potentially captured all of these elements in combination. Indeed, temporal (i.e., order) and relative-preference dependent assumptions on the weights of the sequence-event utilities showed that participants were actually being influence by more than just the average.

Although the relative-preference dependent assumption in particular successfully accounted for participant results, it suffers from important limitations, including forcing a ranking among the individual sequence experiences, and thus perhaps an unnecessarily excessive separation among them, as well as its inability to feasibly scale with larger sequences, as it requires more and more parameterization. We therefore next tested an absolute-preference-dependent model in its place, which does not suffer from the same limitations, and found comparable results between the two types of preference dependency.

Nonetheless, averaging and order-dependency also included in the models continue to make them realistically untenable as they still assume memorization of complete sequences, which at some point is implausible as sequences grow. We next, therefore, examined absolute-preference dependency along with a temporal model based on windowed evaluations (i.e., evaluations conducted within a circumscribed window of time into the past), which can be applied to arbitrary-length sequences. The window-based models successfully explained the retrospective-evaluation results in our experiments, with a combined window evaluation and absolute-preference dependency model performing best.

However, the window-based models would also be expected to have limited generalizability, with three particularly important limitations of their own: an inability to capture (1) primacy effects, or (2) extreme peaks outside of the window (that remain prominent in memory), and (3) having an arbitrary discontinuity at the window boundary. A preference-dependent discounting (PD) model was therefore proposed to compensate for these problems, although by itself, the PD model did not perform as well as the windowed preference-dependent (WP) model. And yet when we compared the models in more detail, we found that they each captured different sequence characteristics (such as the factors the WP cannot explain). We therefore considered a combined WP and PD model and found that it performed better than the individual models alone, although barely beating the WP model (and not significantly). Finally, however, we found that model performance was dependent on the individual differences among the participants. That is, for roughly a quarter of the participants, the combined WP-PD model outperformed the individual ones—and thus with a weighted combined model (Equation 29) needed to subsume all participants.

Of course, with the more sophisticated models, such as the combined WP-PD model, one must consider the extent to which the better performance results from a larger number of parameters, and thus it was important to determine exactly what the PD model contributed to the combined model, which we did by examining whether it in fact reflected actual working memory. First, compared to the simple discounting model, the preference-dependent discounting model showed higher explanatory power particularly for participants with high preference dependency in working memory. This indicates that the model actually simulates the preference-dependent working-memory mechanism in the evaluations. Second, focusing on the discounting rates themselves, we found them to be correlated with the preference-dependent accuracy rates in the working-memory task with length four, simultaneously in three preference levels of the five total in the PD model. Thus, parameters in the PD model, which were meant to represent working memory, actually correlated with preference-dependent working-memory performance of the participants, suggesting that the PD model reflects working-memory features. At the same time, although the results suggest that one's working-memory ability contributes to the discount rate of preference, they do not imply that the preference works exactly like working memory or completely captures it. In any case, correlations between working-memory ability and model parameters are consistent with results of other studies showing that the peak-end rule fails with easy tasks, without distractions or need for high memory capacity, implicating contributions of working memory to the experience evaluation (Langer et al., 2005; Liersch and McKenzie, 2009) (We also note again that the Preference-dependent discounting model can be interpreted as a recurrent network combined with an unsupervised image classification system. In this interpretation, preference-dependence of working-memory ability naturally occurs as a result of variances in weights of summation).

Taken together, the success of the combined WP-PD model shows that, even within our experimental paradigm, multiple features of experienced sequences influence retrospective evaluation. These include having a predominantly window-based evaluation that is also preference-dependent (i.e., most preferred experiences remembered better), with additional effects (e.g., preference-dependence, primacy) outside the main evaluation window. At the same time, our results show that significant individual differences exist in exactly how people evaluate sequences of their experiences retrospectively.

With respect to the individual differences in the study, although we were indeed able to leverage it to evaluate and develop our models, we note that the empirical data nonetheless yielded averages with relatively low standard error, and thus reflected features shared across the participants. Moreover, our major finding showed that we could split the participants into two groups: (1) 22.7% of the participants whose behavior was dominated by windowed evaluation, and (2) the other 77.3% whose behavior required both windowed and discounting models to be explained. Thus, even the individual differences could be classified into two groups, with each group explained well by our models.

The general finding of multiple effects on retrospective evaluation (from specific sequence characteristics to individual differences) shows that comprehensive quantitative analyses are needed to elucidate the factors and the conditions under which they influence retrospective evaluation. We hope our experimental paradigm and mathematical framework help show the success of this approach and how it can be undertaken. As discussed in section “Experiments,” human attractiveness as observed in images is a real and immediately consumable reward, with extensive theoretical and empirical evidence supporting this; and thus we expect our basic findings to generalize to other types of pleasurable stimuli. Future studies are needed to test this prediction. And even though we suspect that the basic findings will generalize from the men to women as well when using a comparable consummatory real-time reward (such as images of men), female participants also need to be tested in the future to verify this.

Future work is indeed necessary on a range of different conditions (Ariely and Carmon, 2000), including types of experiences (besides images of attractiveness), positivity/negativity of experiences, timescale of experiences (Xiaowei et al., 2013), quantitative/qualitative experiences, real/virtual experiences, and individual differences factors such as gender and personality traits (Yoon et al., 2019). Future work is also needed to examine the role of selective attention and other memory mechanisms in retrospective evaluation (Jung et al., 2019).

With respect to the timescale of experience, we designed our tasks in as wide a time range as possible for a highly controlled laboratory situation. We tested sequences that ranged from four to 100 pictures, which we consider a strength of our study. These experiences thus range from seconds to minutes timescales. To test experiences beyond this, like hours or days, it is difficult to provide controlled stimuli and reliable testing conditions to measure the responses quantitatively in the laboratory. In any case, this does imply that studies with such larger timescales are needed for further validation of our model. Indeed, another strength of our computational study was the development of a flexible, robust, and scalable model that should be able to capture these larger timescales.

At the same time, future work can extend ours by dealing with shortcomings that we encountered. These include some limitations in statistical power, such that more participants and trials would help more firmly establish the results. For the windowed evaluation model, many window sizes accumulated near 10, obviously because of the experimental design, in that the sequence-rating-continued-version task queried their sequence ratings with 8–13 intervals, averaging around 10. Future experiments can vary the rating intervals to attempt to decouple actual evaluation windows from the specific task demands (although this also requires more trials, and thus potential fatigue must be monitored). It would be interesting to determine the extent to which the evaluation window (and thus underlying working memory) is affected by external factors, including its pliability based on task demands. Beyond this, the different window-size distributions for the Windowed Average and Windowed Preference-dependent models that we obtained (Figure 8B) suggests that memory works at multiple levels hierarchically (Sakai et al., 2003; Huntley et al., 2011), with each level focusing on a particular sequence size: e.g., level one, of say period 10, whose units are individual events (such as our pictures); level two, whose units consist of each period at the lower level (i.e., sequences of 10 images), and so on. In our paradigm, only two levels of memory were easily captured across all participants (one picture and a period of 8–13 pictures). A future experiment with more participants and a design focused on studying multiple memory levels could attempt to characterize these levels quantitatively; and then the evaluation rule used at each level could also be examined. For example, one could test a model of nested windowed averaging, which averages within each period (lower window), and then takes recent several periods (higher window) to average them *a la* our current Windowed Averaging model. Moreover, one could develop a model that uses utilities of chunks (average of period) for past memories, and utilities of individual events for recent memories. Parameterizing the temporal cutline of past and recent, one could potentially find when the memory chunks are formed. Regarding potential evaluation rules at each memory level, one might imagine that a peak-end type heuristic may be more suitable when evaluating sequences in which their unit events are nested sequences themselves.

The highest correlation-test result in our study was 0.74, in the combined WP-PD model for Group 2 (Figure 13B). Therefore, in principle, roughly 0.26 of correlation value (46% of variance unaccounted for) remains to be explained. Other factors might include other kinds and components of memory, selective attention, and so forth (Ariely and Carmon, 2000).

Brain electrophysiological and imaging experiments can also be conducted using our experimental paradigm and mathematical framework. For example, one could not only test the hypothesis that working memory underlies experience evaluation, but more specifically how it does so, by examining correlations of regional brain activity with specific model parameters.

Finally, comprehensive evaluation models that capture individual retrospective evaluation patterns could potentially be applied in many real-world settings (Nasiry and Popescu, 2011; Hoogerheide and Paas, 2012), helping to improve the quality of life. If we can predict how people evaluate their sequential experience—such as having longer or shorter evaluation windows, the degree of preference dependence, the most impactful order position of peaks—we can design their experience order to optimize their satisfaction. For example, by knowing or being able to predict individual experience-evaluation functions, many real-world cases of sequential events, such as education practices, newspaper articles, television programming, streaming services, shopping venues, websites, trip tour recommendations, and so on could in principle be tailored to optimize sequence preferences. A quantitative characterization of retrospective sequence evaluation might also help to localize dysfunction and design tailored treatments in important relevant cases, ranging from natural aging to addiction. We hope our general paradigm and research findings help point toward these ends.

## Data Availability Statement

Datasets are available upon request: The raw data supporting the conclusions of this article will be made available by the authors, without undue reservation.

## Ethics Statement

The experiments involving human participants were reviewed and approved by the Institutional Review Board of Korea Advanced Institute of Science & Technology. The participants provided their written informed consent to participate in this study.

## Author Contributions

All authors designed the experiment. SL, SY, and JK collected the data. SL analyzed the data. JJ supervised the study. SL and JDK wrote the manuscript. SL, JDK, and JJ revised the manuscript.

## Conflict of Interest

The authors declare that the research was conducted in the absence of any commercial or financial relationships that could be construed as a potential conflict of interest.
