# Supplementary material for: Retrospective Evaluation of Sequential Events and the Influence of Preference-Dependent Working Memory: A Computational Examination
Source: Front Comput Neurosci. 2020 Sep 11;14:65. doi: 10.3389/fncom.2020.00065 (PMC7516338; doi:10.3389/fncom.2020.00065)
Supplement: Supplementary file 1 [file Data_Sheet_1.docx]

Supplementary Material


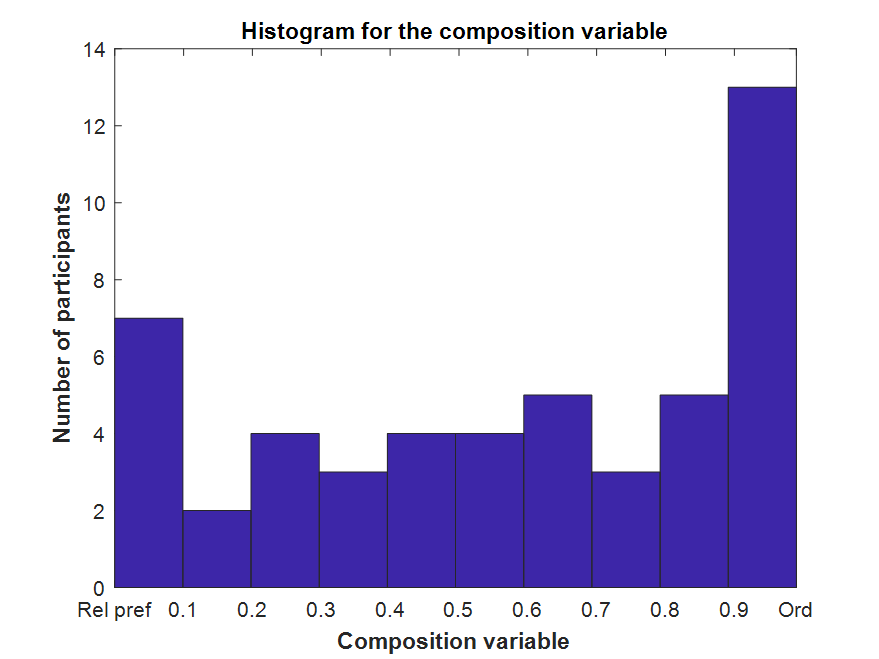


Supplementary Fig 1. Histogram for the composition variable $\boldsymbol{\lambda}$ in the combined model. Mean and standard deviation are 0.5792 and 0.3311. Many participants are distributed at both extremes.


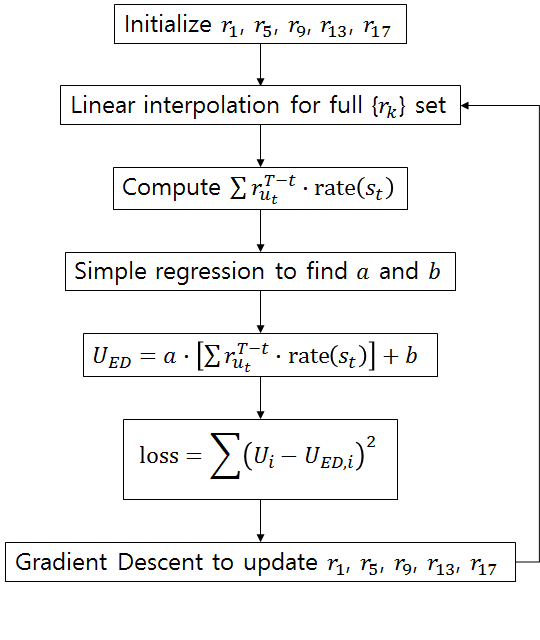


Supplementary Fig 2. Flow chart of the gradient descent algorithm that minimized the sum of squared errors. Using SSE, parameters $\boldsymbol{a}$ and $\boldsymbol{b}$ can be represented in closed forms. Discount rates were computed for only five representatives and the others were defined by linear interpolation. See text for term descriptions.


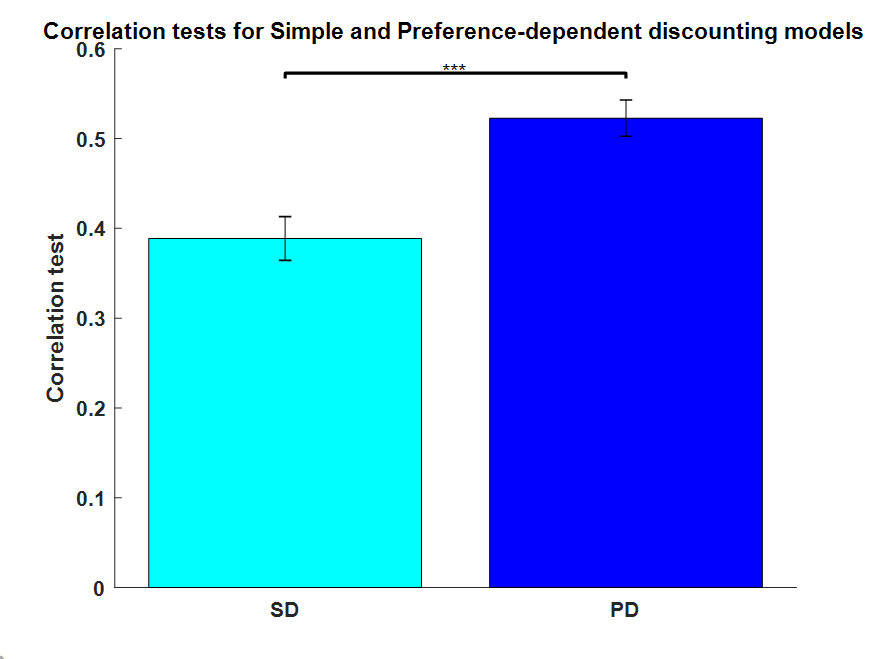


Supplementary Fig 3. Correlation tests for the simple discounting and preference-dependent discounting models. The correlation of the PD model with the empirical data for the sequence-rating-continued-version task was significantly higher than for the SD model, confirming that model training was successful.

**Supplementary Table 1. Pearson’s correlations between the predictions of order-dependent and relative-preference-dependent models in seven types of sequences.** The two models collapse into the same for monotonic sequences.

| Seq. type | Increase | Decrease | Zigzag | Peak1 | Peak2 | Peak3 | Peak4 | Total |
| --- | --- | --- | --- | --- | --- | --- | --- | --- |
| Corr. | 1 | 1 | 0.4305 | 0.8563 | 0.8594 | 0.8099 | 0.8410 | 0.9799 |

**Supplementary Table 2. Correlations between the predictions of the absolute-preference-dependent model and two previous models.** First row shows the correlation between the absolute-preference-dependent model and the order-dependent model. Second row shows the correlation between the absolute-preference-dependent model and the relative-preference-dependent model.

|  | Inc | Dec | Zigzag | Peak1 | Peak2 | Peak3 | Peak4 | Total |
| --- | --- | --- | --- | --- | --- | --- | --- | --- |
| Order | 0.9224 | 0.9148 | 0.3749 | 0.8500 | 0.8474 | 0.8207 | 0.8498 | 0.9658 |
| Rel pref | 0.9224 | 0.9148 | 0.3093 | 0.8446 | 0.8826 | 0.8354 | 0.8314 | 0.9707 |

Supplementary Table 3. Multiple linear regression of discount rates in the SD and PD models with preference-dependent accuracy rates in the working-memory task with length four. Discount rates r1, r5, and r9 of the PD model had significant p-values of 0.0259, 0.0010, and 7.1620e-04, respectively, using the preference-dependent accuracy rates in the working-memory task with length four. The other regression p-values were over 0.05.

| Model | Disc. rate | p-value | R square | w1 | w2 | w3 | w4 |
| --- | --- | --- | --- | --- | --- | --- | --- |
| SD | r | 0.1508 | 0.0320 | -0.2835 | 0.3326 | 0.0880 | -0.3597 |
| PD | r1 | 0.0259 | 0.1095 | 0.9509 | -2.0086 | 0.5019 | 1.3098 |
| PD | r5 | 0.0010 | 0.2570 | 0.0676 | 2.1575 | -0.4433 | -2.0937 |
| PD | r9 | 0.0007 | 0.2665 | -0.6082 | 1.5386 | 0.0012 | -1.8738 |
| PD | r13 | 0.0594 | 0.0857 | -0.3591 | 0.9128 | -0.3272 | -0.5125 |
| PD | r17 | 0.0590 | 0.0546 | -0.2268 | -0.2311 | -0.2466 | -0.0953 |

**Supplementary Table 4.** Linear regression of discount rates in the SD and PD models with preference-dependent accuracy rates in the working-memory task with length seven. Discount rate r17 of the PD model had a significance p-value of 0.0329 using the preference-dependent accuracy rates in the working-memory task with length seven. The other regressions had p-values over 0.05.

| Model | Disc. rate | p-val. | R^2^ | w1 | w2 | w3 | w4 | w5 | w6 | w7 |
| --- | --- | --- | --- | --- | --- | --- | --- | --- | --- | --- |
| SD | r | 0.053 | 0.062 | 0.031 | -0.046 | 0.009 | 0.018 | -0.047 | 0.023 | 0.029 |
| PD | r1 | 0.057 | 0.094 | 0.043 | -0.064 | 0.017 | 0.025 | -0.068 | 0.022 | 0.040 |
| PD | r5 | 0.065 | 0.090 | -0.076 | -0.005 | 0.190 | 0.016 | -0.092 | -0.350 | 0.134 |
| PD | r9 | 0.079 | 0.084 | -0.034 | 0.016 | 0.089 | 0.017 | -0.058 | -0.035 | -0.046 |
| PD | r13 | 0.059 | 0.059 | -0.000 | 0.005 | -0.026 | -0.004 | 0.013 | 0.056 | -0.005 |
| PD | r17 | 0.033 | 0.075 | 0.014 | -0.009 | -0.024 | -0.005 | 0.018 | -0.013 | 0.021 |
